# Theoretical Study of the Reaction Mechanism of Phenol–Epoxy Ring-Opening Reaction Using a Latent Hardening Accelerator and a Reactivity Evaluation by Substituents

**DOI:** 10.3390/molecules28020694

**Published:** 2023-01-10

**Authors:** Ryusuke Mitani, Hidetoshi Yamamoto, Michinori Sumimoto

**Affiliations:** Graduate School of Sciences and Technology for Innovation, Yamaguchi University, 2-16-1 Tokiwadai, Ube 755-8611, Yamaguchi, Japan

**Keywords:** density functional theory, phenol–epoxy ring-opening reaction, latent hardening accelerator, tetraphenylphosphonium-tetraphenylborate

## Abstract

The mechanism of the phenol–epoxide ring-opening reaction using tetraphenylphosphonium-tetraphenylborate (TPP-K) was investigated using the density functional theory (DFT) method. The reaction was initiated by breaking the P-B bond of TPP-K. The generated tetraphenylborate (TetraPB^−^) reacted with phenol to form a phenoxide ion, which combined with tetraphenylphosphonium (TPP^+^) to produce the active species, i.e., tetraphenylphosphonium phenolate (TPP-OPh). The phenoxide ion in TPP-OPh nucleophilically attacked the epoxide. Simultaneously, the H atom in the phenolic OH group moved to the O atom of the ring-opened epoxide. The formed phenoxide ion bound to TPP^+^ again, and TPP-OPh was regenerated. The rate-determining steps in the reaction were the cleavage of the P-B bond and the triphenylborane-forming reaction. The free energies of activation were calculated to be 36.3 and 36.1 kcal/mol, respectively. It is also suggested that these values in the rate-determining steps could be manipulated by substituents introduced on the Ph group of TetraPB^−^. Based on these results, it is possible to construct new design guidelines for latent hardening accelerators such as TPP-K.

## 1. Introduction

Phenol–epoxy thermosetting resins are functional materials that exhibit excellent heat resistance, chemical resistance, and electrical properties. Owing to these properties, they have been used in a wide range of materials in recent years, including materials used in electronic devices, composites, coatings, and adhesives as well as packaging materials in the electronics industry [1,2,3,4,5,6,7,8,9]. Although phenol–epoxy thermosetting resins are excellent functional materials, their curing temperatures during molding are extremely high [10]. To address this issue, these reactions have added hardeners or hardening accelerators that can lower the activation free energy of the reaction and progress the reaction easily. Therefore, various hardeners and hardening accelerators for the phenol–epoxy opening reaction have been studied and developed through experiments and theoretical calculations [7,8,11,12,13,14,15,16]. Various types of hardening accelerators have been widely used, such as thiols, imidazole, tertiary amines, and phosphorus compounds. Recently, Truong et al. clarified the mechanism of the phenol–epoxide ring-opening reaction catalyzed by amines and phosphines [11]. Through theoretical calculations, we previously described the reaction mechanism and reactivity of the hardening accelerator in the phenol–epoxy ring-opening reaction using imidazole compounds [10]. It has been reported that, in these mechanisms, the ring-opening reaction proceeds via the nucleophilic attack of an epoxide by amines, phosphines, and imidazole. Furthermore, we proposed that it is possible to manipulate the reactivity through investigations of the orbital energies of the highest occupied molecular orbitals (HOMOs) in nucleophiles.

However, because the addition of a hardening accelerator rapidly causes a curing reaction in which a phenolic resin and an epoxy resin react to give a hardened material, it is difficult to store the raw material for a long duration. To solve this problem, a latent hardening accelerator with high long-term stability at room temperature in raw materials has been developed as a curing agent [7,8,17,18,19,20,21,22,23,24,25,26]. A latent hardening accelerator initiates the reaction by applying an external stimulus such as heat or light; therefore, the curing reaction does not proceed unless these stimuli are applied. Therefore, long-term storage becomes possible. Tetraphenylphosphonium-tetraphenylborate (TPP-K), which is a phosphorus-based curing accelerator, is used as a latent hardening accelerator for phenol–epoxy resins. In TPP-K, tetraphenylphosphonium (TPP^+^) and tetraphenylborate (TetraPB^−^) are bound by an ionic bond. However, the role of TPP-K in the curing reaction and its reaction mechanism have not yet been clarified.

Theoretical calculations are crucial for investigating chemical reactions without preceding experiments and have been used to analyze the mechanisms of organic and inorganic reactions. Transition state (TS) searches help clarify the reaction mechanisms in detail as well as determine the reason for the high selectivity for the target. Many theoreticians have used this method to analyze reactions that have already been examined and yielded the proposed products in experiments [27,28,29,30,31,32,33,34,35,36].

The purpose of this work was to clarify the detailed mechanism of the phenol–epoxide ring-opening reaction using TPP-K as a latent hardening accelerator and to elucidate the effect of the latent hardening accelerator on the reaction. Through an investigation of these problems using theoretical calculations, we aimed to construct a design guide for new latent hardening accelerators. In our calculation, we used ethylene oxide (EP) as the epoxide.

## 2. Results and Discussion

### 2.1. Geometry Changes in Phenol–Epoxy Ring-Opening Reaction Using TPP-K

**Scheme 1:**
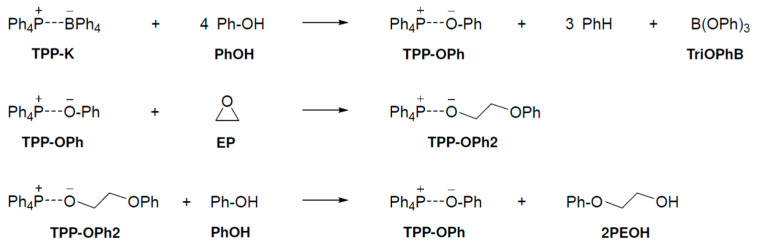
Reaction mechanism proposed by experimental chemists.

We examined the pathway by which three compounds (PhOH, EP, and TPP-K) are involved in the reaction at the same time at which the TPP-K-catalyzed epoxide ring opening reacts with phenol. The curing reaction mechanism of PhOH and EP with TPP-K is shown in Scheme 1. In the first step of the proposed reaction mechanism, TPP-K reacts with four PhOH molecules to produce tetraphenylphosphonium phenolate (TPP-OPh), benzene, and triphenylborate (TriOPhB). The generated TPP-OPh opens the ring of EP to form TPP-OPh2, which includes EP in the system. Finally, PhOH reacts with TPP-OPh2 to produce 2-phenoxyethanol (2PEOH) and regenerate TPP-OPh. We investigated the reaction mechanism according to the proposed mechanism.

**Figure 1:**
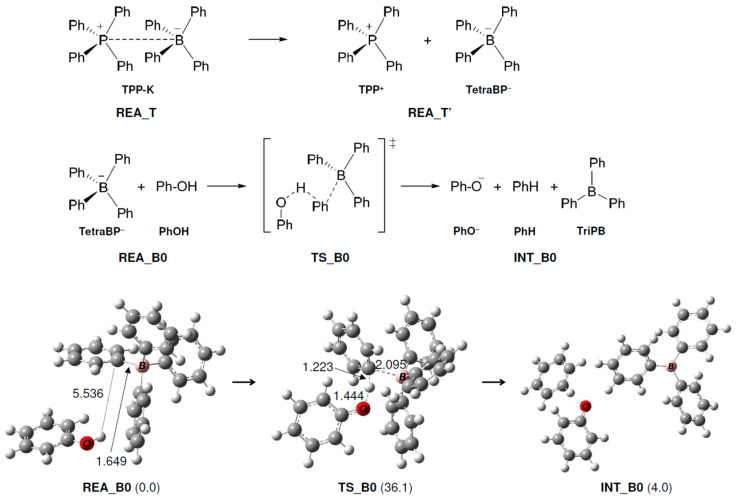
Geometry and free energy changes in the triphenylborane-forming reaction. The bond lengths are in Å. Relative free energies (kcal/mol) to REA_B0 are in parentheses.

In this reaction, the ionic bond of P-B in TPP-K is broken, and it is thought that the reaction starts when TPP^+^ and TetraPB^−^ are generated. We calculated the dissociation free energy of TPP-K to be 36.3 kcal/mol. In Path A1, the TetraPB^−^ generated by decomposition reacts with PhOH. The calculated reaction mechanisms, geometry changes, and relative Gibbs free energies for these steps are shown in Figure 1. In TS and TS_B0, the H atom of the OH group in PhOH approaches the C atom of the phenyl group in TetraPB^−^ to form triphenylborane (TriPB), a phenoxide ion (PhO^−^), and benzene. The activation free energy (Δ*G*^‡^) was estimated to be 36.1 kcal/mol.

**Figure 2:**
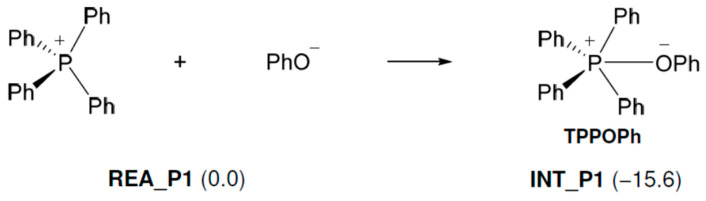
Free energy changes in the tetraphenylphosphonium phenolate-forming reaction. Relative free energies (kcal/mol) to REA_P1 are in parentheses.

**Figure 3:**
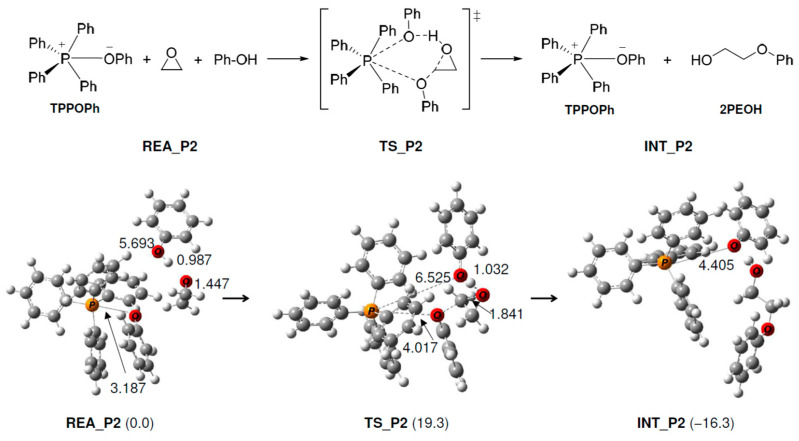
Geometry and free energy changes in the phenol–epoxy ring-opening reaction using TPP-OPh. The bond lengths are in Å. Relative free energies (kcal/mol) to REA_P2 are in parentheses.

As shown in Figure 2, the following reaction occurs in TPP^+^. First, the produced PhO^−^ (Figure 1) approaches TPP^+^ and reacts with it to produce TPP-OPh. This reaction proceeds without an activation barrier and is exothermic (15.6 kcal/mol). Next, the O atom of PhO^−^ in TPP-OPh approaches the C atom of EP and performs a nucleophilic attack on EP, as shown in Figure 3. At the same time, the O-C bond in EP breaks, and the H atom of the OH group in PhOH moves to the O atom of EP, producing 2PEOH, and regenerates TPP-OPh without the formation of TPP-OPh2. The Δ*G*^‡^ and Δ*G* values are 19.3 and −16.3 kcal/mol, respectively.

**Figure 4:**
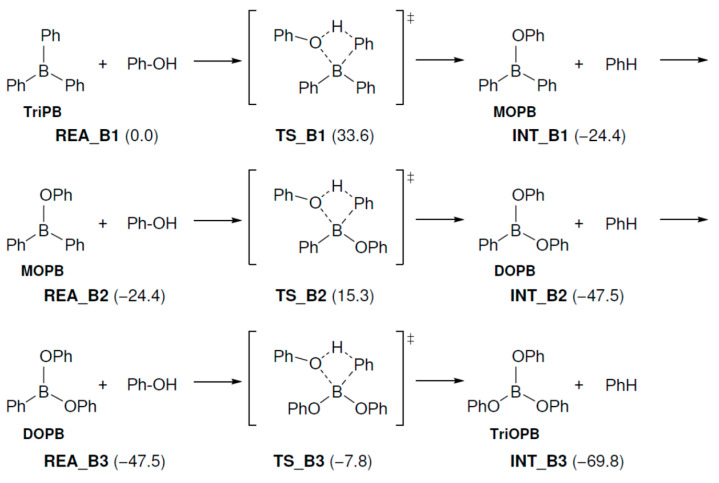
Geometry and free energy changes in the triphenylborate-forming reaction. The bond lengths are in Å. Relative free energies (kcal/mol) to REA_B1 are in parentheses.

The generated TriPB sequentially reacts with three molecules of PhOH to produce TriOPhB in three steps. The relative energies of the reaction mechanisms are specified in Figure 4. The Δ*G*^‡^ values are 33.6, 39.7, and 39.7 kcal/mol, respectively. Although these Δ*G*^‡^ values are high, these reactions are expected to proceed because they are performed at a high temperature of over 100 °C. However, it remains unclear how the boron compounds produced by these reactions are used in the present reaction mechanism. This will be studied in a future investigation.

### 2.2. Free Energy Changes in the Predicted Reaction Mechanism

**Figure 5:**
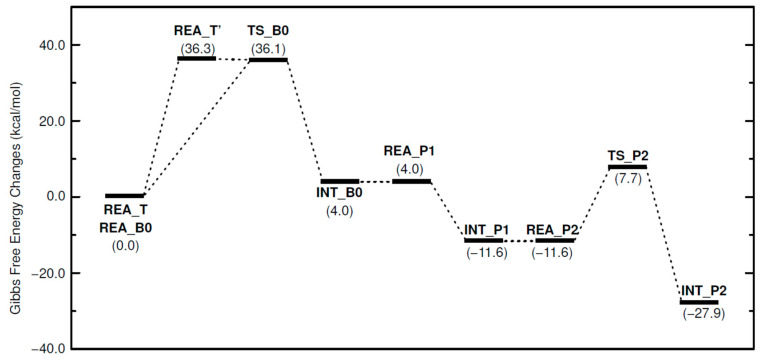
Free energy changes in phenol–epoxide ring-opening reaction using TPP-K. Relative free energies (kcal/mol) to REA_T and REA_B0 are in parentheses.

Figure 5 shows the relative free energies of the calculated reaction pathways. In the phenol–epoxide ring-opening reaction with TPP-K starting from REA_T, the P-B ionic bond dissociation energy was calculated to be 36.3 kcal/mol. The triphenylborane-forming reaction between one Ph group of the generated TetraPB^−^ and the H atom of the OH group in PhOH proceeds following the dissociation of TPP-K. The Δ*G*^‡^ value for this reaction was calculated to be 36.1 kcal/mol to produce PhO^−^, benzene, and TriPB. The generated PhO^−^ approaches TPP^+^ produced by the dissociation of TPP-K and produces TPP-OPh without an activation barrier (REA_P1 → INT_P1). The stabilization energy of this reaction is 15.6 kcal/mol. After TPP-OPh is produced, the epoxide-opening reaction with phenol proceeds using PhO^−^ in TPP-OPh. The Δ*G*^‡^ values were estimated to be 19.3 kcal/mol. The TPP-OPh thus produced becomes the active species, and the reaction is repeated while consuming EP and PhOH. In the four-step reaction, the formation of INT_B0 is a slightly endothermic reaction (4.0 kcal/mol). However, all other elementary processes are exothermic. The rate-determining step in these reaction mechanisms is the P-B ionic bond dissociation reaction of TPP-K or the triphenylborane-forming reaction between TetraPB^−^ and PhOH.

These results indicate that the reaction is initiated by the dissociation of TPP-K. The generated TetraPB^−^ reacts with phenol to form phenoxide ions. The phenoxide ion approaches TPP^+^, another compound produced by the dissociation of TPP-K, and readily generates TPP-OPh. Through the binding of TPP^+^ to the phenoxide ion, the highly reactive phenoxide ions remain stable in the reaction system. After TPP-OPh is produced, the phenoxide ion in TPP-OPh becomes a nucleophilic species, and an epoxide-opening reaction occurs. The reaction using the phenoxide ion in TPP-OPh as the nucleophilic species proceeds easily and repeatedly. The important functions of TPP-K clarified in this study are as follows: No reaction occurs unless TPP-K dissociates. TetraPB^−^ produced by dissociation forms a highly nucleophilic phenoxide ion. The generated TPP^+^ combines with the phenoxide ion to form the active species. As a result, TPP-K becomes a latent curing accelerator, and the phenol–epoxide ring-opening reaction occurs more easily than without a catalyst.

### 2.3. Reactivity Evaluation by Introduction of Substituents

To clarify which factor affects the functionality of the latent hardener, we investigated the Δ*G*^‡^ and binding energy (BE) values based on the effects of substituents in BPh_4_^−^, as shown in Table 1. The F group was introduced as an electron-withdrawing substituent and the OMe group was introduced as an electron-donating substituent at the ortho- or meta-position of the Ph group in the BPh_4_^−^ compounds. When the F group was used as an electron-withdrawing substituent, the P-B binding energy was weaker than that of the H group. It is presumed that the electron density of the B atom decreases and the P-B ionic bond is weakened by the effect of the electron-withdrawing substituent. In the triphenylborane-forming reaction, electrons are deprived of the electron system of the Ph group, which deactivates the system and reduces the reactivity. The Δ*G*^‡^ values of these reactions with the introduction of electron-withdrawing substituents are approximately 10 kcal/mol higher than those of BPh_4_^−^. Conversely, when the OMe group was introduced as an electron-donating group, the P-B binding energy was almost the same as that of the H group. However, the Δ*G*^‡^ value of the triphenylborane-forming reaction decreased, and the reactivity improved. This was probably because the electron-donating substituent activated the electron system of the Ph group. Next, we considered the relationship between the binding energy and the Δ*G*^‡^ value of the triphenylborane-forming reaction. In the case of compounds in which electron-withdrawing substituents are introduced, the P-B bond is cleaved first when the temperature in the reaction system increases. The Δ*G*^‡^ values for the triphenylborane-forming reaction between B(C_6_H_4_F)_4_^−^ and phenol are about 13.6−15.4 kcal/mol higher than the bond dissociation energy. Therefore, it is expected that this reaction does not occur for some time after the P-B bond breaks. In contrast, in the case of compounds with electron-donating substituents, the Δ*G*^‡^ values for the triphenylborane-forming reaction between B(C_6_H_4_OMe)_4_^−^ and phenol are approximately the same as the bond dissociation energy. Therefore, it is considered that the triphenylborane-forming reaction proceeds simultaneously when the P-B bond is broken. Based on the above considerations, it is suggested that the P-B bond energy and Δ*G*^‡^ values for the triphenylborane-forming reaction, which is the initiation reaction, can be manipulated by introducing substituents into the Ph group of BPh_4_^−^. Based on these results, it is possible to construct new design guidelines for latent hardening accelerators such as TPP-K.

## 3. Materials and Methods

All geometry optimizations and energy changes were calculated using DFT, where the B3PW91 functional [37,38] was used for the exchange-correlation term. We ascertained that each equilibrium structure exhibited no imaginary frequencies and each TS had only one imaginary frequency. The intrinsic reaction coordinate (IRC) calculation [39] was performed from the TS structure, the reactants and products were searched, and these were connected to form a reaction pathway. In these calculations, 6-311+G(d) basis sets were employed for C, O, and N atoms [40,41], whereas the 6-31G(d,p) basis set was used for H atoms. The relative energies of all the reactions were calculated using the Gibbs free energies for each optimized geometry. Coordinates and free energy values of the optimized structures are given in Table S1 of ESI. All calculations were performed using the Gaussian 09 program package [42].

## 4. Conclusions

In the present study, we theoretically investigated the mechanism of the phenol–epoxide ring-opening reaction using TPP-K. We clarified the role and design guidelines for the latent hardening accelerator in this reaction. The important results are summarized as follows:

(1) The phenol–epoxy ring-opening reaction using TPP-K proceeds via four main elementary reaction processes.
(2) After the P-B bond in TPP-K is cleaved, the reaction between BPh_4_^−^ and phenol occurs.
(3) The rate-determining steps in the reaction are the cleavage of the P-B bond and the triphenylborane-forming reaction. The Δ*G*^‡^ values are 36.3 and 36.1 kcal/mol, respectively.
(4) TPP-OPh, which is generated by the combination of TPP^+^ and phenoxide ions, is the active species.
(5) By introducing electron-withdrawing or electron-donating substituents to the Ph group in BPh_4_^−^, it is possible to manipulate the P-B bond energy and Δ*G*^‡^ value of the triphenylborane-forming reaction between BPh_4_^−^ and phenol.

From our calculations, the mechanism of the phenol–epoxide curing reaction using TPP-K and the function of TPP-K were clarified. In the future, design guidelines can be proposed for the latent hardening accelerators in this reaction.

## Figures and Tables

**Table 1:** Substituents introduced into the Ph group in TetraPB^−^, binding energy (BE) of the P-B bond, and Δ*G*^‡^ values in triphenylborane-forming reaction.

Substituent	BE (kcal/mol)	Δ*G*^‡^ (kcal/mol)
H	36.3	36.1
*m*-F	32.9	48.3
*p*-F	32.7	46.3
*m*-OMe	36.9	35.3
*p*-OMe	36.2	32.2

## Data Availability

Not applicable.

## Supplementary Materials

The following supporting information can be downloaded at https://www.mdpi.com/article/10.3390/molecules28020694/s1, Table S1: Cartesian coordinates of all structures calculated at the B3PW91 level.
