# Supplementary material for: Theoretical Study of the Reaction Mechanism of Phenol–Epoxy Ring-Opening Reaction Using a Latent Hardening Accelerator and a Reactivity Evaluation by Substituents
Source: Molecules. 2023 Jan 10;28(2):694. doi: 10.3390/molecules28020694 (PMC9861531; doi:10.3390/molecules28020694)
Supplement: Supplementary file 1 [file molecules-28-00694-s001.zip › molecules-2093697-supplementary.pdf]

Supplementary Materials

1. Cartesian coordinates calculated at the B3PW91 level

Table S1. Cartesian coordinates of all structuers calculated at the B3PW91 level. Free energy values are in hartree. Imazinary frequency values are in cm^{-1}.

TPP-K

Sum of electronic and thermal Free Energies: -2218.402960

P	3.28358200	-0.02530300	-0.01617300
C	2.42662000	-1.57502500	-0.36480800
C	3.43211400	0.22618800	1.77167000
C	4.94846400	-0.15986100	-0.72896200
C	2.44801300	1.40736500	-0.72445000
C	1.05517200	-1.58798400	-0.63465400
C	3.38084000	-0.86300400	2.64487700
C	5.09436800	-0.67291300	-2.02413800
C	1.23317200	1.83793100	-0.17187400
C	3.14743800	-2.77723600	-0.30165500
C	3.63868500	1.51810000	2.26792400
C	6.07008000	0.27257400	-0.01791500
C	3.00664300	2.07759100	-1.81917600
C	0.40378100	-2.80036700	-0.83809200
C	3.54875100	-0.65900500	4.00939100
C	6.35625600	-0.74161800	-2.60089300
C	0.57773300	2.92580100	-0.73595400
C	2.48729300	-3.98141400	-0.50643800
C	3.80664400	1.71100600	3.63364200
C	7.33011400	0.19689000	-0.60272200
C	2.34400300	3.17055300	-2.36394000
C	1.11919600	-3.99115700	-0.77243000
C	3.76405100	0.62428900	4.50259400
C	7.47314500	-0.30641800	-1.89146900
C	1.13024400	3.58920700	-1.82735000
H	0.48346700	-0.66989000	-0.68361000
H	3.19018000	-1.86026500	2.26665700
H	4.22902500	-1.02451800	-2.57497900
H	0.78877900	1.34517200	0.68841800
H	4.21434600	-2.77353500	-0.10763300
H	3.64892600	2.36895600	1.59612500
H	5.96475200	0.65744200	0.98950400
H	3.95226700	1.75942100	-2.24035100
H	-0.66037600	-2.80773400	-1.04755000
H	3.49714500	-1.50225900	4.68799400
H	6.46723200	-1.13949300	-3.60278600
H	-0.37541200	3.23182200	-0.32479900
H	3.04265500	-4.91133300	-0.46166100
H	3.95719000	2.71237600	4.01955800
H	8.19966800	0.52818100	-0.04726700
H	2.77663000	3.69108100	-3.21051300
H	0.60334100	-4.93095400	-0.93159000
H	3.88570400	0.78017200	5.56840200
H	8.45666600	-0.36499000	-2.34344900
H	0.60515500	4.43073200	-2.26391200
C	-3.24597600	-1.51554400	-0.59046700
C	-2.31597800	0.46948300	1.06722600
C	-4.94781400	0.01731300	0.70152900
C	-3.42220600	1.12517400	-1.25344900
C	-3.34805200	-2.60772800	0.29482900
C	-1.41380100	-0.40016400	1.70028600
C	-5.18877900	0.40458800	2.02706200
C	-4.47548800	2.00045100	-1.56095000
C	-3.05315600	-1.85351400	-1.94024300
C	-2.24835200	1.81429500	1.48522900
C	-6.07246700	-0.42354200	-0.02089900
C	-2.26936900	1.26738300	-2.04698500
C	-3.23832600	-3.93112400	-0.12283900
C	-0.52891400	0.02387500	2.69521400
C	-6.46304200	0.36734000	2.59686700
C	-4.39512400	2.93913100	-2.59040800
C	-2.95540300	-3.17640800	-2.37959000
C	-1.37418400	2.25531600	2.47518100
C	-7.34903900	-0.46124300	0.52989800
C	-2.17560300	2.18770000	-3.08772800
C	-3.03843400	-4.22708800	-1.47124000
C	-0.50482800	1.35694200	3.09458400
C	-7.55336300	-0.06301500	1.85013900
C	-3.24587100	3.03483100	-3.36806700
H	-3.55959500	-2.41224800	1.34239400
H	-1.41445500	-1.45096000	1.42887300
H	-4.36116500	0.74345300	2.64246000
H	-5.38989200	1.94550500	-0.97913000
H	-3.00393600	-1.06246800	-2.68133300
H	-2.92393800	2.53244100	1.02828900
H	-5.93862900	-0.75291800	-1.04794200
H	-1.41385600	0.62647200	-1.84626600
H	-3.33563200	-4.73579000	0.60060300
H	0.12952200	-0.69600100	3.17383600
H	-6.60059800	0.67484700	3.62981400
H	-5.23890400	3.59396500	-2.78828300
H	-2.82790600	-3.38359500	-3.43855300
H	-1.38099100	3.29904400	2.77772300
H	-8.18707900	-0.80705000	-0.06853600
H	-1.26967800	2.24160400	-3.68575700
H	-2.97733300	-5.25728100	-1.80844100
H	0.16479400	1.68813700	3.88192700
H	-8.54596600	-0.09348500	2.28827500
H	-3.18542000	3.75262300	-4.18021700
B	-3.46894600	0.02209800	-0.02941300

PhOH

Sum of electronic and thermal Free Energies: -307.359182

C	-0.22067500	-1.21961300	0.00009500
C	-0.93840500	-0.02458400	0.00035300
C	-0.26324300	1.19537900	0.00005300
C	1.12839900	1.21505300	-0.00005200
C	1.85187900	0.02751200	0.00004600
C	1.16754100	-1.18645600	-0.00014900
H	-0.76330000	-2.15717400	-0.00017100
H	-0.82149600	2.12747500	-0.00008000
H	1.64547800	2.16819100	-0.00017300
H	2.93510900	0.04639900	-0.00000100
H	1.71969500	-2.11977200	-0.00025200
O	-2.29874500	-0.11022000	-0.00005800
H	-2.67849800	0.77289700	-0.00094700

TPP-OPh

Sum of electronic and thermal Free Energies:-1574.174528

P	-0.83181000	1.46536300	-0.22577000
C	-0.80556700	3.30042600	-0.47588800
C	-0.12722200	4.15172300	0.40302900
C	-1.48971000	3.86266000	-1.56041300
C	-0.10914500	5.52444500	0.18331100
H	0.36169900	3.73757200	1.27299400
C	-1.50938700	5.24048800	-1.74970000
H	-2.00050100	3.22731100	-2.27293800
C	-0.80721100	6.07461900	-0.88650000
H	0.43571700	6.16578500	0.86685900
H	-2.06194400	5.65734700	-2.58425900
H	-0.80738400	7.14746900	-1.04437600
C	-2.42972300	0.65892700	0.17563500
C	-3.63536500	1.20150000	-0.26968400
C	-2.44073900	-0.50647100	0.94357200
C	-4.84043700	0.58187200	0.04685000
H	-3.64379200	2.11052300	-0.86019300
C	-3.64584500	-1.13432200	1.23823000
H	-1.51168200	-0.91191000	1.32587000
C	-4.84752600	-0.59210400	0.79221300
H	-5.77331000	1.01970800	-0.29037400
H	-3.64520800	-2.04073800	1.83312300
H	-5.78662300	-1.07636200	1.03515500
C	0.72567000	0.49794200	0.01526400
C	1.84605500	1.03835600	0.64920600
C	0.78901100	-0.80423500	-0.48622100
C	3.01308300	0.29134300	0.76428900
H	1.80639000	2.03470200	1.06539100
C	1.94387800	-1.56406800	-0.32924900
H	-0.05851300	-1.23053100	-1.01006200
C	3.06299400	-1.01488800	0.28671300
H	3.88140200	0.72865900	1.24463600
H	1.97044800	-2.58060000	-0.70562500
H	3.96972200	-1.60005600	0.39249000
C	-0.84152300	1.06987000	-2.10793400
C	0.22914600	1.55593000	-2.87352000
C	-1.78480600	0.26717200	-2.75252300
C	0.34402300	1.26821600	-4.22636100
H	0.99034300	2.17181000	-2.40356700
C	-1.67387800	-0.02823700	-4.11318900
H	-2.62032800	-0.14415700	-2.19943900
C	-0.61323400	0.47264200	-4.85498200
H	1.18205100	1.66111300	-4.79209000
H	-2.42287900	-0.65388700	-4.58715100
H	-0.52657600	0.24440700	-5.91162600
O	-0.61323400	1.74294800	1.67356700
C	-1.45364600	2.14196200	2.63765200
C	-2.52020800	3.03524100	2.44661900
C	-1.22885300	1.64950400	3.93598100
C	-3.32750500	3.41032200	3.51729500
H	-2.71326500	3.44347900	1.46327700
C	-2.03528300	2.03619900	4.99650200
H	-0.40451300	0.96058600	4.08303100
C	-3.09595200	2.91945600	4.79740200
H	-4.14702400	4.10054200	3.34322700
H	-1.83643700	1.64167500	5.98794800
H	-3.72808400	3.21874000	5.62553200

TriOPhB

Sum of electronic and thermal Free Energies: -945.241808

B	-0.09132600	-1.24118800	0.58937400
O	-0.60627500	-2.43842200	0.99522000
O	-0.27189700	-0.82117700	-0.69687900
C	-0.50524400	-2.98221500	2.25511300
C	-0.88725800	-2.27639600	3.39165100
C	-0.07308100	-4.30090200	2.35197300
C	-0.81883200	-2.89782900	4.63431900
H	-1.22529100	-1.25164600	3.30393300
C	-0.01066900	-4.91239500	3.59924000

H	0.20525500	-4.83111400	1.44911000
C	-0.38096500	-4.21419600	4.74500700
H	-1.11271800	-2.34637000	5.52045900
H	0.32780800	-5.93984600	3.67302900
H	-0.33165800	-4.69274200	5.71612200
C	-0.97240800	-1.49056800	-1.67397000
C	-2.30141800	-1.86131100	-1.49618300
C	-0.32751300	-1.72312600	-2.88416000
C	-2.98017700	-2.48396500	-2.53867100
H	-2.79383400	-1.67070300	-0.55081600
C	-1.01637000	-2.34469900	-3.91992900
H	0.70355900	-1.41124300	-2.99977600
C	-2.34345100	-2.72916800	-3.75170600
H	-4.01485500	-2.77657600	-2.39893200
H	-0.51191000	-2.52667200	-4.86230800
H	-2.87851800	-3.21330300	-4.56031200
C	1.20103700	0.74279100	1.16411000
C	0.42565100	1.82571100	0.76294600
C	1.03695400	3.05423700	0.53502500
C	2.40920600	3.20500000	0.71203600
H	-0.64173900	1.70371200	0.62569100
H	0.43356200	3.89779200	0.21876900
H	2.87944200	4.16514100	0.53446800
O	0.63238300	-0.47604500	1.45816300
C	2.57178800	0.88244300	1.35164400
H	3.14942700	0.02584300	1.67774500
C	3.17235600	2.11565400	1.12212500
H	4.24146600	2.22355900	1.26708900

EP
Sum of electronic and thermal Free Energies: -153.743592

C	-0.66064300	-0.48658500	0.00000000
C	0.00000000	0.82004900	0.00000000
O	0.75870600	-0.38328000	0.00000000
H	-1.09774700	-0.86434100	0.92077100
H	-1.09774700	-0.86434100	-0.92077100
H	0.04485000	1.39707200	0.92022800
H	0.04485000	1.39707200	-0.92022800

TPP-OPh2
Sum of electronic and thermal Free Energies: -1727.935249

P	0.09306400	0.02053300	-0.08319400
C	-0.03391900	-0.02334800	1.85797300
C	1.16687300	-0.02578900	2.58348800
C	-1.22438500	-0.00883500	2.58783500
C	1.18041400	-0.02246700	3.97163900
H	2.11488200	-0.02972200	2.05269300
C	-1.22013800	-0.00465700	3.98527700
H	-2.17881500	0.00330100	2.07448000
C	-0.01967400	-0.01333200	4.68188200
H	2.12663700	-0.02560200	4.50254300
H	-2.16222100	0.00671300	4.52373600
H	-0.01412700	-0.01038400	5.76643400
C	-1.72815100	-0.04429300	-0.36200200
C	-2.40471000	1.12053900	-0.73295100
C	-2.43945900	-1.24037000	-0.25581800
C	-3.77587100	1.09124100	-0.96679100
H	-1.85893400	2.05004500	-0.84907300
C	-3.80936200	-1.26919200	-0.50708300
H	-1.93160100	-2.15830500	0.01730000
C	-4.48263200	-0.10312500	-0.85456100
H	-4.29107100	2.00417700	-1.24502300
H	-4.34867300	-2.20681600	-0.42848700
H	-5.55002700	-0.12523600	-1.04363700
C	1.03943700	-1.58028000	-0.11202400
C	2.08771200	-1.77585100	-1.01656100
C	0.71591000	-2.61558400	0.77282200
C	2.80683000	-2.96717200	-1.01826200
H	2.32259700	-1.00563300	-1.73808000
C	1.40687700	-3.82207300	0.73861400
H	-0.06610600	-2.47741200	1.50915800
C	2.46374000	-3.99687700	-0.14906200
H	3.62660800	-3.09426100	-1.71646000
H	1.12888800	-4.61774500	1.42089000
H	3.01559800	-4.93036700	-0.16307400
C	0.91966700	1.68576800	-0.02628800
C	0.43948900	2.65925700	0.85453700
C	2.01358400	1.99560900	-0.84073100
C	1.02501300	3.92057900	0.90569800
H	-0.39120100	2.43697600	1.51305100
C	2.62573400	3.24125000	-0.75338800
H	2.37037700	1.27110000	-1.55773900
C	2.12915700	4.21099400	0.11227300
H	0.62492500	4.66864200	1.58115700
H	3.48447600	3.45773900	-1.37935400
H	2.59931900	5.18676800	0.16683700
C	3.72377400	-0.55665000	-5.43792600
C	2.35924500	-0.24503300	-5.42751300
C	1.77981100	0.34473100	-6.55317500
C	2.56788800	0.61392400	-7.67202000
C	3.92142800	0.30581600	-7.68564200
C	4.49326300	-0.28293600	-6.55738200
H	0.72781500	0.59617900	-6.57142800
H	2.10703600	1.07210800	-8.54052400
H	4.52484100	0.51871600	-8.56007800
H	5.54907700	-0.53124400	-6.55017300

O	1.68867600	-0.55490200	-4.28921700
O	0.30951600	0.19693800	-1.88464400
C	-0.22242900	-0.65879400	-2.85507100
C	0.29796200	-0.25951600	-4.21835400
H	-1.32089000	-0.59059500	-2.89496200
H	0.03287600	-1.71379200	-2.67144600
H	0.13474000	0.81410100	-4.36952800
H	-0.24088900	-0.81918100	-4.99531700
H	4.15751300	-1.01549000	-4.55738800

1P2OH
Sum of electronic and thermal Free Energies: -461.116803

C	1.29463600	-1.28405700	-0.15326500
C	0.47851100	-0.14858800	-0.13064100
C	1.04978600	1.11272700	0.04382900
C	2.43235100	1.22509400	0.19480400
C	3.24660700	0.10148100	0.17306200
C	2.66592200	-1.15521800	-0.00284300
H	0.44090200	2.00721500	0.06063200
H	2.86805000	2.20912600	0.32974100
H	4.31929200	0.19891100	0.29075200
H	3.28806500	-2.04328000	-0.02171900
O	-0.85247900	-0.37705400	-0.29240000
O	-3.59561900	-0.62701900	0.67354100
C	-3.16071200	0.20987800	-0.37679500
C	-1.74149100	0.71860900	-0.22338500
H	-3.81918000	1.08384500	-0.49384700
H	-3.23457900	-0.38804700	-1.28739100
H	-1.62495700	1.24319200	0.73716700
H	-1.53241600	1.43886000	-1.02760700
H	0.82865500	-2.25274500	-0.28820900
H	-3.54270500	-0.14404100	1.50276200

TPP^+
Sum of electronic and thermal Free Energies: -1267.270250

P	3.34319900	-0.02409500	0.00088300
C	2.46586400	-1.56210500	-0.36868400
C	3.45334200	0.22643500	1.78877000
C	4.99587800	-0.15029000	-0.72360000
C	2.45754600	1.39006700	-0.69680400
C	1.12619600	-1.54326500	-0.76707600
C	3.24363400	-0.83442000	2.67457400
C	5.13318500	-0.68567600	-2.01178500
C	1.31787900	1.87223300	-0.03796600
C	3.14115200	-2.78193900	-0.22392400
C	3.78371500	1.49788200	2.27814600
C	6.11955500	0.30661500	-0.02898100
C	2.87913300	1.97847500	-1.89280700
C	0.46434600	-2.74183800	-1.01063800
C	3.37460000	-0.62418500	4.04293900
C	6.38973100	-0.74714000	-2.60034300
C	0.60174400	2.92827800	-0.58673800
C	2.46866100	-3.97272700	-0.46650900
C	3.91535400	1.69490200	3.64673000
C	7.37297800	0.23654400	-0.62704400
C	2.15575000	3.03713200	-2.43091500
C	1.13182400	-3.95272800	-0.85745200
C	3.71307700	0.63499300	4.52770200
C	7.50759500	-0.28444600	-1.90995900
C	1.01859900	3.50820800	-1.78269100
H	0.60380200	-0.60378200	-0.90118500
H	2.96684800	-1.81478900	2.30601400
H	4.26934500	-1.06390800	-2.54740800
H	0.99725000	1.43678200	0.90212000
H	4.18692800	-2.80401000	0.06260000
H	3.92487400	2.33203200	1.59958700
H	6.02478200	0.70158500	0.97526700
H	3.77139800	1.62702600	-2.39666000
H	-0.57208600	-2.72701400	-1.32595700
H	3.20576300	-1.44514100	4.72949900
H	6.49676000	-1.16231400	-3.59543700
H	-0.27802800	3.30253500	-0.07686900
H	2.99053000	-4.91597800	-0.35708300
H	4.16892000	2.67784900	4.02560900
H	8.24480800	0.58474400	-0.08620700
H	2.48625600	3.49709900	-3.35451600
H	0.61246700	-4.88413200	-1.05054700
H	3.81230700	0.79437600	5.59508000
H	8.48681000	-0.33820600	-2.37122800
H	0.45933100	4.33490700	-2.20494400

$TetraBP^-$
Sum of electronic and thermal Free Energies: -951.075688

B	-0.00000700	0.00000000	0.00001700
C	-0.97624700	1.32751600	0.11063700
C	-0.79525400	2.36988600	1.03226300
C	-2.07289700	1.47326100	-0.76037300
C	-1.63440400	3.48418600	1.08000000
H	0.02210200	2.30745000	1.74388800
C	-2.91660400	2.58031600	-0.73056800
H	-2.27637900	0.68301400	-1.47814200
C	-2.70118300	3.60024600	0.19464000
H	-1.45426600	4.26281600	1.81701800
H	-3.74912400	2.64536300	-1.42654600
H	-3.35778000	4.46484000	0.22811700
C	0.97656200	-0.11038400	1.32729800

C	0.79624200	-1.03227100	2.36954700
C	2.07288400	0.76107100	1.47290500
C	1.63568100	-1.07982200	3.48363700
H	-0.02081100	-1.74424800	2.30718500
C	2.91687600	0.73144800	2.57974500
H	2.27586000	1.47905300	0.68271900
C	2.70211100	-0.19403000	3.59957100
H	1.45605600	-1.81705100	4.26218700
H	3.74912700	1.42775700	2.64469600
H	3.35894300	-0.22736600	4.46399300
C	-0.97628300	-1.32753000	-0.11015700
C	-0.79580000	-2.36983200	-1.03195300
C	-2.07247400	-1.47332000	0.76142900
C	-1.63497700	-3.48412800	-1.07931200
H	0.02115700	-2.30732900	-1.74402700
C	-2.91619900	-2.58037000	0.73200500
H	-2.27558300	-0.68311300	1.47934800
C	-2.70128400	-3.60023600	-0.19339300
H	-1.45523900	-4.26271000	-1.81648000
H	-3.74835900	-2.64545500	1.42841200
H	-3.35791100	-4.46481900	-0.22658000
C	0.97594400	0.11041000	-1.32773200
C	0.79483900	1.03198100	-2.37013100
C	2.07243900	-0.76076800	-1.47366300
C	1.63374500	1.07952000	-3.48462300
H	-0.02241000	1.74371500	-2.30756000
C	2.91590100	-0.73116000	-2.58090900
H	2.27600300	-1.47851300	-0.68341400
C	2.70038000	0.19401100	-3.60085300
H	1.45353100	1.81650100	-4.26327100
H	3.74832100	-1.42724500	-2.64608800
H	3.35678300	0.22733600	-4.46560100

PhO^-
Sum of electronic and thermal Free Energies: -306.807298

O	-2.34285800	-0.00001000	0.00026000
C	-1.07726200	-0.00003400	-0.00008400
C	-0.28723200	1.20867600	-0.00022800
C	-0.28718300	-1.20869600	-0.00022500
C	1.09830400	1.19749300	-0.00006100
H	-0.83053800	2.15077400	0.00014400
C	1.09836700	-1.19745400	-0.00008200
H	-0.83035600	-2.15086000	0.00020400
C	1.82558100	0.00002700	-0.00008500
H	1.63457500	2.14656800	0.00022500
H	1.63461800	-2.14653700	0.00022200
H	2.91110700	0.00006100	0.00171000

PhH
Sum of electronic and thermal Free Energies: -232.143049

C	-1.32288800	-0.43472000	0.00000100
C	-0.28483900	-1.36284800	0.00001300
C	1.03792200	-0.92815600	-0.00000200
C	1.32287000	0.43477200	0.00000300
C	0.28489200	1.36283600	0.00000500
C	-1.03795800	0.92811600	-0.00001000
H	-2.35302900	-0.77352400	-0.00000600
H	-0.50667100	-2.42436500	-0.00001600
H	1.84634100	-1.65097000	-0.00003100
H	2.35305500	0.77344200	-0.00001300
H	0.50659300	2.42438100	0.00001400
H	-1.84628600	1.65103200	-0.00000500

TriPB
Sum of electronic and thermal Free Energies: -719.478487

B	-0.00056300	-0.00104100	-0.00016800
C	0.50100300	1.48181200	-0.00178600
C	-0.21084500	2.49604500	0.66491400
C	1.68353800	1.85559000	-0.66663700
C	0.24200500	3.81043300	0.68322900
H	-1.12592100	2.24345100	1.19041100
C	2.12407300	3.17415300	-0.68020500
H	2.25683900	1.10025600	-1.19400300
C	1.40752300	4.15356300	0.00278500
H	-0.31600100	4.56946600	1.22100600
H	3.02918800	3.43864400	-1.21641800
H	1.75611600	5.18082900	0.00449600
C	1.03295900	-1.17666500	0.00133700
C	0.76364200	-2.38938700	-0.65962100
C	2.27069200	-1.06419300	0.66131400
C	1.68699300	-3.42865000	-0.67675600
H	-0.17996300	-2.51064300	-1.18139300
C	3.18454100	-2.11179200	0.67585500
H	2.51095500	-0.14428900	1.18410300
C	2.89732400	-3.29424200	-0.00129100
H	1.46192000	-4.34621100	-1.20978100
H	4.12375000	-2.00552100	1.20810000
H	3.61427000	-4.10836100	-0.00230500
C	-1.53568000	-0.30703800	-0.00024600
C	-2.06024300	-1.42736900	0.67027500
C	-2.44866500	0.52745200	-0.67104600
C	-3.42505400	-1.69198100	0.68597200
H	-1.38584800	-2.09145700	1.20090700
C	-3.81087100	0.24973800	-0.68745600
H	-2.07952200	1.39933700	-1.20108900
C	-4.30297900	-0.85723200	-0.00075500
H	-3.80494700	-2.55229400	1.22657500
H	-4.49078200	0.89927700	-1.22829500
H	-5.36696500	-1.06869500	-0.00075300

TPPOPh
Sum of electronic and thermal Free Energies: -1574.174528

P	-0.83181000	1.46536300	-0.22577000
C	-0.80556700	3.30042600	-0.47588800
C	-0.12722200	4.15172300	0.40302900
C	-1.48971000	3.86266000	-1.56041300
C	-0.10914500	5.52444500	0.18331100
H	0.36169900	3.73757200	1.27299400
C	-1.50938700	5.24048800	-1.74970000
H	-2.00050100	3.22731100	-2.27293800
C	-0.80721100	6.07461900	-0.88650000
H	0.43571700	6.16578500	0.86685900
H	-2.06194400	5.65734700	-2.58425900
H	-0.80738400	7.14746900	-1.04437600
C	-2.42972300	0.65892700	0.17563500
C	-3.63536500	1.20150000	-0.26968400
C	-2.44073900	-0.50647100	0.94357200
C	-4.84043700	0.58187200	0.04685000
H	-3.64379200	2.11052300	-0.86019300
C	-3.64584500	-1.13432200	1.23823000
H	-1.51168200	-0.91191000	1.32587000
C	-4.84752600	-0.59210400	0.79221300
H	-5.77331000	1.01970800	-0.29037400
H	-3.64520800	-2.04073800	1.83312300
H	-5.78662300	-1.07636200	1.03515500
C	0.72567000	0.49794200	0.01526400
C	1.84605500	1.03835600	0.64920600
C	0.78901100	-0.80423500	-0.48622100
C	3.01308300	0.29134300	0.76428900
H	1.80639000	2.03470200	1.06539100
C	1.94387800	-1.56406800	-0.32924900
H	-0.05851300	-1.23053100	-1.01006200
C	3.06299400	-1.01488800	0.28671300
H	3.88140200	0.72865900	1.24463600
H	1.97044800	-2.58060000	-0.70562500
H	3.96972200	-1.60005600	0.39249000
C	-0.84152300	1.06987000	-2.10793400
C	0.22914600	1.55593000	-2.87352000
C	-1.78480600	0.26717200	-2.75252300
C	0.34402300	1.26821600	-4.22636100
H	0.99034300	2.17181000	-2.40356700
C	-1.67387800	-0.02823700	-4.11318900
H	-2.62032800	-0.14415700	-2.19943900
C	-0.61323400	0.47264200	-4.85498200
H	1.18205100	1.66111300	-4.79209000
H	-2.42287900	-0.65388700	-4.58715100
H	-0.52657600	0.24440700	-5.91162600
O	-0.61323400	1.74294800	1.67356700
C	-1.45364600	2.14196200	2.63765200
C	-2.52020800	3.03524100	2.44661900
C	-1.22885300	1.64950400	3.93598100
C	-3.32750500	3.41032200	3.51729500
H	-2.71326500	3.44347900	1.46327700
C	-2.03528300	2.03619900	4.99650200
H	-0.40451300	0.96058600	4.08303100
C	-3.09595200	2.91945600	4.79740200
H	-4.14702400	4.10054200	3.34322700
H	-1.83643700	1.64167500	5.98794800
H	-3.72808400	3.21874000	5.62553200

MOPB
Sum of electronic and thermal Free Energies: -794.732828

C	-0.02370700	1.25950900	-0.08882400
C	1.01284500	1.67108100	-0.94202500
C	-0.58333600	2.22307800	0.76524800
C	1.46200700	2.98653100	-0.95049000
H	1.47411600	0.95525800	-1.61421900
C	-0.11807300	3.53438500	0.78171900
H	-1.38840500	1.93976700	1.43595000
C	0.90231700	3.92022300	-0.08213100
H	2.25547900	3.28266400	-1.62804600
H	-0.55648500	4.25636400	1.46225600
H	1.26011500	4.94416400	-0.07973200
C	-2.08493800	-0.56774200	-0.05030700
C	-2.50628800	-1.85884200	0.31065000
C	-3.07342200	0.36992200	-0.38839500
C	-3.85369300	-2.19453300	0.34777300
H	-1.75974300	-2.60340000	0.56505900
C	-4.42380500	0.03618600	-0.36612700
H	-2.78012200	1.37105300	-0.68629500
C	-4.81603600	-1.24581600	0.00760800
H	-4.15710000	-3.19490100	0.63736900
H	-5.17012200	0.77406100	-0.64009100
H	-5.86871600	-1.50690500	0.03104700
B	-0.56219400	-0.21380400	-0.09070400
O	0.27352400	-1.30468000	-0.14438400
C	1.63742300	-1.33409700	-0.00176200
C	2.38671300	-1.96119300	-0.99342700
C	2.25589200	-0.82972800	1.13890900
C	3.76612900	-2.06324300	-0.84972000
H	1.87943100	-2.36304700	-1.86261900
C	3.63612800	-0.93822000	1.27215100

H	1.65873000	-0.35686400	1.90916300
C	4.39691600	-1.55163100	0.28113800
H	4.34833200	-2.54954400	-1.62458600
H	4.11707000	-0.54251600	2.15976700
H	5.47177600	-1.63493100	0.39163400

DOPB
Sum of electronic and thermal Free Energies: -869.987548

C	-1.15782800	-1.52181200	-0.07660900
C	-2.39544700	-1.12163100	-0.60263100
C	-1.06393600	-2.81170000	0.46975600
C	-3.49403100	-1.97417100	-0.58258900
H	-2.50688800	-0.13628700	-1.04034900
C	-2.16361500	-3.66084500	0.50732600
H	-0.11199400	-3.14878600	0.86488800
C	-3.38221800	-3.24263800	-0.02060200
H	-4.43904400	-1.64752200	-1.00257300
H	-2.07030800	-4.65050600	0.94118500
H	-4.24115700	-3.90473700	0.00234000
B	0.11913100	-0.62353900	-0.11677300
O	0.16434200	0.73926600	-0.26036400
C	-0.84405900	1.65111700	-0.10041700
C	-1.09105300	2.54898600	-1.13518400
C	-1.55541500	1.73536500	1.09382700
C	-2.07185900	3.52228500	-0.97813600
H	-0.51106400	2.47430000	-2.04725300
C	-2.53563600	2.71140300	1.23844000
H	-1.34420100	1.03717000	1.89514700
C	-2.79945100	3.60678000	0.20594200
H	-2.26449300	4.21967800	-1.78577300
H	-3.09200900	2.77282200	2.16707600
H	-3.56176800	4.36773600	0.32519600
C	2.55365900	-0.64706700	0.00913300
C	3.43919700	-0.85238500	-1.04337000
C	2.93219500	0.10680100	1.11548300
C	4.70895500	-0.28690000	-0.99121300
H	3.12506200	-1.45412700	-1.88789600
C	4.20364900	0.66878400	1.15754200
H	2.23526100	0.24791000	1.93285400
C	5.09575200	0.47641600	0.10651000
H	5.39830900	-0.44684600	-1.81282400
H	4.49744500	1.25822700	2.01896800
H	6.08612700	0.91491500	0.14467600
O	1.32714700	-1.26913800	-0.03601700

TriOPB
Sum of electronic and thermal Free Energies: -945.241808

B	-0.09132600	-1.24118800	0.58937400
O	-0.60627500	-2.43842200	0.99522000
O	-0.27189700	-0.82117700	-0.69687900
C	-0.50524400	-2.98221500	2.25511300
C	-0.88725800	-2.27639600	3.39165100
C	-0.07308100	-4.30090200	2.35197300
C	-0.81883200	-2.89782900	4.63431900
H	-1.22529100	-1.25164600	3.30393300
C	-0.01066900	-4.91239500	3.59924000
H	0.20525500	-4.83111400	1.44911000
C	-0.38096500	-4.21419600	4.74500700
H	-1.11271800	-2.34637000	5.52045900
H	0.32780800	-5.93984600	3.67302900
H	-0.33165800	-4.69274200	5.71612200
C	-0.97240800	-1.49056800	-1.67397000
C	-2.30141800	-1.86131100	-1.49618300
C	-0.32751300	-1.72312600	-2.88416000
C	-2.98017700	-2.48396500	-2.53867100
H	-2.79383400	-1.67070300	-0.55081600
C	-1.01637000	-2.34469900	-3.91992900
H	0.70355900	-1.41124300	-2.99977600
C	-2.34345100	-2.72916800	-3.75170600
H	-4.01485500	-2.77657600	-2.39893200
H	-0.51191000	-2.52667200	-4.86230800
H	-2.87851800	-3.21330300	-4.56031200
C	1.20103700	0.74279100	1.16411000
C	0.42565100	1.82571100	0.76294600
C	1.03695400	3.05423700	0.53502500
C	2.40920600	3.20500000	0.71203600
H	-0.64173900	1.70371200	0.62569100
H	0.43356200	3.89779200	0.21876900
H	2.87944200	4.16514100	0.53446800
O	0.63238300	-0.47604500	1.45816300
C	2.57178800	0.88244300	1.35164300
H	3.14942700	0.02584300	1.67774500
C	3.17235600	2.11565400	1.12212500
H	4.24146600	2.22355900	1.26708900

REA_BO
Sum of electronic and thermal Free Energies: -1258.429963

C	-5.49126000	1.89768800	0.97309400
C	-3.48868100	1.11809700	-0.69200500
C	-6.01698300	0.12279700	-0.91363800
C	-4.37378500	-0.56869100	1.14268400
C	-6.08885300	2.96742000	0.27923700
C	-2.99735400	2.43189100	-0.71791000
C	-5.85605200	-0.02667100	-2.29897000
C	-4.99221900	-1.82457000	1.23096200
C	-5.52354800	1.99156400	2.37202800
C	-2.72590800	0.17115700	-1.40182900
C	-7.30032300	-0.17569300	-0.41662100
C	-3.28234600	-0.36724500	2.01190000
C	-6.65902800	4.05888800	0.92678700
C	-1.83773900	2.78520800	-1.41028200
C	-6.89024800	-0.45708500	-3.13226100
C	-4.58262300	-2.80109900	2.14301800
C	-6.09732400	3.07624500	3.03849600
C	-1.57262700	0.50712200	-2.10509600
C	-8.34044000	-0.61218500	-1.23155400
C	-2.86174600	-1.32515300	2.92894800
C	-6.66615200	4.12175400	2.31966300
C	-1.11861000	1.82520900	-2.11433200
C	-8.14045700	-0.75883200	-2.60336000
C	-3.51809500	-2.55486300	3.00842800
H	-6.11449100	2.93363600	-0.80675100
H	-3.53321200	3.20541600	-0.17672600
H	-4.89618000	0.20861700	-2.74771600
H	-5.82533400	-2.04909000	0.57264400
H	-5.09497600	1.19066100	2.96647200
H	-3.04854800	-0.86688100	-1.40034000
H	-7.49083300	-0.04642700	0.64568000
H	-2.74796200	0.57778400	1.96332400
H	-6.10135700	3.09969200	4.12525700
H	-1.02508800	-0.26112000	-2.64422300
H	-9.31291300	-0.83077200	-0.79811100
H	-2.02156500	-1.11654900	3.58539100
B	-4.84142000	0.64149000	0.12169600
O	-1.99021800	-4.70256200	1.07012700
H	-2.54056700	-3.94668600	1.33114900
C	-0.84617200	-4.27982400	0.47941500
C	0.09302400	-5.25176400	0.12564200
C	-0.58536300	-2.93349900	0.21468300
C	1.28320500	-4.87520600	-0.48402500
H	-0.12838300	-6.29168900	0.33627900
C	0.61135100	-2.57088800	-0.39413300
H	-1.31478900	-2.17525200	0.48187800
C	1.55313100	-3.53367200	-0.74767600
H	2.00549000	-5.63844500	-0.75581400
H	0.79632800	-1.52187500	-0.59735600
H	2.48245000	-3.24339100	-1.22472200
H	-0.21812100	2.09579500	-2.65782100
H	-7.11400300	4.96769000	2.83276900
H	-3.20821300	-3.30147000	3.73341100
H	-8.94810000	-1.09438100	-3.24720700
H	-1.49585400	3.81695200	-1.39670200
H	-7.10653900	4.86046400	0.34494600
H	-5.10522200	-3.75340800	2.18383400
H	-6.71589500	-0.55303400	-4.20085100

TS_BO
Imaginary frequency value: -593.3
Sum of electronic and thermal Free Energies: -1258.372428

C	-5.38400900	1.36426900	0.86236300
C	-3.27358200	1.62303200	-0.80669500
C	-5.09581800	-0.40912000	-1.13768800
C	-3.17574400	-0.53943000	1.13759900
C	-6.64699300	1.73362100	0.36440900
C	-3.44233200	3.01451600	-0.71301100
C	-4.82428700	-0.46813600	-2.51213400
C	-3.91319400	-1.41339300	1.95883500
C	-5.08923300	1.79200000	2.16538800
C	-2.15029400	1.17536200	-1.52932100
C	-6.12285500	-1.24868200	-0.67264300
C	-2.17200500	0.22595500	1.76604300
C	-7.55452900	2.48062100	1.11277800
C	-2.55660300	3.91203600	-1.30713900
C	-5.51736300	-1.31971600	-3.36790800
C	-3.69920500	-1.48965100	3.32922700
C	-5.99178800	2.52889900	2.92820500
C	-1.25576100	2.06663000	-2.11672600
C	-6.81329200	-2.11647100	-1.51291400
C	-1.93701200	0.14539800	3.13341500
C	-7.23329400	2.88080100	2.40595000
C	-1.45410200	3.44159200	-2.01286300
C	-6.51159100	-2.15676200	-2.87146100
C	-2.70957400	-0.70737100	3.92155100
H	-6.92582800	1.43098900	-0.63958400
H	-4.29002400	3.40862200	-0.16226400
H	-4.04938300	0.16687700	-2.92534500
H	-4.65145100	-2.06135700	1.49945900
H	-4.12977800	1.54061800	2.60106500
H	-1.96469500	0.10840100	-1.61643100
H	-6.40454300	-1.20760500	0.37496800
H	-1.56399900	0.88823700	1.15861400
H	-5.72216800	2.82904400	3.93676600
H	-0.39729800	1.68121200	-2.65932200
H	-7.59159800	-2.75727000	-1.10877500
H	-1.15551000	0.74647400	3.58846100
B	-4.35349000	0.62196400	-0.14753500
O	-2.27041300	-1.87504900	-0.94357500
H	-2.87274900	-1.02878000	0.05889400
C	-1.88197800	-3.06998700	-0.59916400
C	-2.19264300	-4.19593700	-1.40831000
C	-1.12274300	-3.32962300	0.57323000
C	-1.76290500	-5.47196800	-1.07566300

H	-2.78123400	-4.02079700	-2.30279100
C	-0.71013500	-4.61532600	0.90003500
H	-0.86403100	-2.49352800	1.21403100
C	-1.01793500	-5.70387500	0.08348400
H	-2.01798000	-6.30469100	-1.72711300
H	-0.13112600	-4.77033400	1.80744700
H	-0.68766000	-6.70453800	0.34217100
H	-0.75694600	4.13559000	-2.47307600
H	-7.93903000	3.45559700	2.99790600
H	-2.53374300	-0.76938800	4.99113700
H	-7.04684400	-2.83010600	-3.53406900
H	-2.72813800	4.98073000	-1.21318200
H	-8.51676600	2.74519700	0.68365100
H	-4.28750600	-2.17065900	3.93610800
H	-5.27321000	-1.33507400	-4.42607600

INT_B0
Sum of electronic and thermal Free Energies: -1258.418877

C	2.48220200	4.02312900	-1.60846900
C	3.94830400	1.94492400	-0.66631600
C	4.92638900	4.47939600	-0.53288900
C	10.25795400	-2.26877800	-0.96831600
C	1.88670100	5.22223700	-1.18014700
C	2.81989000	1.12558200	-0.45432900
C	5.81715400	4.21346200	0.52508400
C	11.19610300	-3.02657700	-0.26831600
C	1.85331900	3.33683900	-2.66174400
C	5.21237400	1.33013300	-0.57111500
C	5.12091300	5.67732500	-1.24841100
C	10.09478600	-2.49257600	-2.33492100
C	0.71580100	5.70321900	-1.75781400
C	2.95383700	-0.22163900	-0.14234800
C	6.85586400	5.08101300	0.84403400
C	11.95922600	-3.99258900	-0.92099000
C	0.70111000	3.82860000	-3.26770400
C	5.36074200	-0.01774000	-0.26033800
C	6.16023200	6.54739600	-0.94040800
C	10.85422600	-3.45716400	-2.99391300
C	0.12376500	5.01058300	-2.81078400
C	4.22351000	-0.79152800	-0.04254900
C	7.02999300	6.24891300	0.10704300
C	11.78930800	-4.20995500	-2.28671600
H	2.34771600	5.78046400	-0.37156800
H	1.82695800	1.56099700	-0.51027200
H	5.70801200	3.30663900	1.10833600
H	11.32923400	-2.85510200	0.79477300
H	2.28498600	2.40592700	-3.01490100
H	6.11105400	1.91302500	-0.73617100
H	4.45778400	5.91565700	-2.07407600
H	9.36733400	-1.90425300	-2.88458000
H	0.26930100	6.62315000	-1.39384700
H	2.06996700	-0.82728600	0.03462400
H	7.54308400	4.81782400	1.64134400
H	12.68795200	-4.57644100	-0.36669300
H	0.24758800	3.28549900	-4.09074300
H	6.36925400	-0.41356300	-0.13957400
H	6.29887200	7.45408900	-1.52121300
H	10.71916800	-3.62290700	-4.05854000
H	-0.78201000	5.38981400	-3.27307600
H	4.32730100	-1.84065400	0.21721600
H	7.85031300	6.92008900	0.34091500
H	12.38342300	-4.96160800	-2.79734400
B	3.79625200	3.47317600	-0.93271700
O	8.42773700	-0.13158400	0.46583500
H	9.66008200	-1.50883600	-0.45727800
C	8.74950700	0.83813800	1.23334800
C	9.83520300	1.73192700	0.94221200
C	8.05474500	1.12546300	2.45714200
C	10.18253600	2.77695300	1.78375100
H	10.38947500	1.55871100	0.02381900
C	8.41549900	2.17722700	3.28658400
H	7.22709600	0.47401500	2.72430800
C	9.48359800	3.02476400	2.97116800
H	11.01965500	3.41820200	1.51240300
H	7.85682900	2.34037900	4.20696700
H	9.76930800	3.83718100	3.63178200

REA_P1
Sum of electronic and thermal Free Energies: -1574.149723

P	8.67327600	-5.02618800	6.83961900
C	10.24910100	-5.64759600	6.19525900
C	7.47152700	-6.37803500	6.94104000
C	9.00211200	-4.38195500	8.50049000
C	8.01267500	-3.74904500	5.77212100
C	10.63116400	-5.35973600	4.88226100
C	7.87092000	-7.71754600	6.92581700
C	10.13023500	-3.57572900	8.70298200
C	7.37283300	-4.12173100	4.57860700
C	11.08641200	-6.41152400	7.01990500
C	6.11172800	-6.05411500	7.04748500
C	8.13332000	-4.64883100	9.56195100
C	8.20979300	-2.39451800	6.07270300
C	11.83765300	-5.84910300	4.39320000
C	6.91759700	-8.72377700	7.03157100
C	10.37194900	-3.03152100	9.95816600
C	6.95559900	-3.13465000	3.69432500
C	12.28685400	-6.89963100	6.51961000
C	5.16728600	-7.06775300	7.15554600
C	8.38792800	-4.10423800	10.81548600
C	7.78124600	-1.42168300	5.17900700
C	12.66117900	-6.62083300	5.20706300
C	5.56840600	-8.40072900	7.15019000
C	9.50222500	-3.29427000	11.01310700
C	7.16039200	-1.78514500	3.98220200
H	10.00012200	-4.74396200	4.25214300
H	8.91688400	-7.97797100	6.81638200
H	10.81673200	-3.37304700	7.88845300
H	7.19959600	-5.16755900	4.34681700
H	10.81293300	-6.60818200	8.05096600
H	5.79179600	-5.01825100	7.02779400
H	7.26648500	-5.28241500	9.41686700
H	8.68644400	-2.09503800	6.99835600
H	12.13593800	-5.61651500	3.37783300
H	7.22969400	-9.76141800	7.01314200
H	11.23978400	-2.40068600	10.10998400
H	6.46979100	-3.41970200	2.76768700
H	12.93567400	-7.48802500	7.15799400
H	4.11652300	-6.81411700	7.23300400
H	7.71328800	-4.31316600	11.63739000
H	7.93469700	-0.37088300	5.39963700
H	13.60265800	-6.99632500	4.82290100
H	4.82809100	-9.18861900	7.22790500
H	9.69481800	-2.86780500	11.99075700
H	6.87862500	-0.99569800	3.27817400
O	7.20299800	0.80542500	2.40913100
C	7.94855600	1.32287000	3.30294400
C	7.42657500	2.09642400	4.39769700
C	9.37459400	1.13777200	3.32839200
C	8.23477600	2.57314300	5.41798500
H	6.35727100	2.28801200	4.40387600
C	10.16639900	1.62362800	4.35757000
H	9.81686400	0.58646000	2.50324500
C	9.61546600	2.33828500	5.42723200
H	7.78572300	3.14803900	6.22539700
H	11.24043900	1.45005100	4.32791400
H	10.24223600	2.72586100	6.22306200

INT_P1
Sum of electronic and thermal Free Energies: -1574.174528

P	-0.83181000	1.46536300	-0.22577000
C	-0.80556700	3.30042600	-0.47588800
C	-0.12722200	4.15172300	0.40302900
C	-1.48971000	3.86266000	-1.56041300
C	-0.10914500	5.52444500	0.18331100
H	0.36169900	3.73757200	1.27299400
C	-1.50938700	5.24048800	-1.74970000
H	-2.00050100	3.22731100	-2.27293800
C	-0.80721100	6.07461900	-0.88650000
H	0.43571700	6.16578500	0.86685900
H	-2.06194400	5.65734700	-2.58425900
H	-0.80738400	7.14746900	-1.04437600
C	-2.42972300	0.65892700	0.17563500
C	-3.63536500	1.20150000	-0.26968400
C	-2.44073700	-0.50647100	0.94357200
C	-4.84043700	0.58187200	0.04685000
H	-3.64379200	2.11052300	-0.86019300
C	-3.64584500	-1.13432200	1.23823000
H	-1.51168200	-0.91191000	1.32587000
C	-4.84752600	-0.59210400	0.79221300
H	-5.77331000	1.01970800	-0.29037400
H	-3.64520800	-2.04073800	1.83312300
H	-5.78662300	-1.07636200	1.03515500
C	0.72567000	0.49794200	0.01526400
C	1.84605500	1.03835600	0.64920600
C	0.78901100	-0.80423500	-0.48622100
C	3.01308300	0.29134300	0.76428900
H	1.80639000	2.03470200	1.06539100
C	1.94387800	-1.56406800	-0.32924900
H	-0.05851300	-1.23053100	-1.01006200
C	3.06299400	-1.01488800	0.28671300
H	3.88140200	0.72865900	1.24463600
H	1.97044800	-2.58060000	-0.70562500
H	3.96972200	-1.60005600	0.39249000
C	-0.84152300	1.06987000	-2.10793400
C	0.22914600	1.55593000	-2.87352000
C	-1.78480600	0.26717200	-2.75252300
C	0.34402300	1.26821600	-4.22636100
H	0.99034300	2.17181000	-2.40356700
C	-1.67387800	-0.02823700	-4.11318900
H	-2.62032800	-0.14415700	-2.19943900
C	-0.61323400	0.47264200	-4.85498200
H	1.18205100	1.66111300	-4.79209000
H	-2.42287900	-0.65388700	-4.58715100
H	-0.52657600	0.24440700	-5.91162600
O	-0.61323400	1.74294800	1.67356700
C	-1.45364600	2.14196200	2.63765200
C	-2.52020800	3.03524100	2.44661900
C	-1.22885300	1.64950400	3.93598100
C	-3.32750500	3.41032200	3.51729500
H	-2.71326500	3.44347900	1.46327700
C	-2.03528300	2.03619900	4.99650200
H	-0.40451300	0.96058600	4.08303100

C	-3.09595200	2.91945600	4.79740200
H	-4.14702400	4.10054200	3.34322700
H	-1.83643700	1.64167500	5.98794800
H	-3.72808400	3.21874000	5.62553200

REA_P2
Sum of electronic and thermal Free Energies: -2035.263541

P	0.00000000	0.00000000	0.00000000
C	0.00000000	0.00000000	1.81463000
C	1.21018000	0.00000000	2.51853300
C	-1.22178700	-0.01348400	2.49941900
C	1.19724100	-0.00096700	3.90828900
H	2.15749300	-0.00729200	1.99388400
C	-1.21382900	-0.01648200	3.89035800
H	-2.15149900	-0.00169500	1.92396100
C	-0.01359000	-0.00687900	4.59420500
H	2.13449400	0.00136700	4.45284200
H	-2.15656400	-0.01822000	4.42561600
H	-0.02021100	-0.00255400	5.67858100
C	1.72534300	-0.31190500	-0.52725100
C	2.13759300	-1.59061400	-0.90895000
C	2.65111100	0.73891400	-0.51762500
C	3.46231700	-1.81587800	-1.27139200
H	1.42544300	-2.40685200	-0.93533100
C	3.97321200	0.50613100	-0.87654600
H	2.33727600	1.73909600	-0.24045700
C	4.38011800	-0.77122500	-1.25355300
H	3.77312700	-2.80948400	-1.57324200
H	4.68363200	1.32482800	-0.86853800
H	5.41072500	-0.94938100	-1.53913600
C	-0.39666300	1.61789200	-0.70367400
C	-0.05697700	1.87652800	-2.03719300
C	-0.99791700	2.60777900	0.07502000
C	-0.33534800	3.12061600	-2.59042600
H	0.43477700	1.12125000	-2.64043800
C	-1.24637400	3.85810900	-0.47974500
H	-1.28387900	2.41694800	1.09938000
C	-0.92544500	4.11139300	-1.80971500
H	-0.08154600	3.31689700	-3.62582100
H	-1.70369500	4.61892100	0.14100400
H	-1.13251300	5.08498600	-2.24032300
C	-0.98756100	-1.37336700	-0.62724600
C	-1.07660100	-2.54531300	0.13292800
C	-1.56616400	-1.31472700	-1.89751400
C	-1.72820300	-3.65537800	-0.38957200
H	-0.65193500	-2.58739200	1.12936700
C	-2.21917500	-2.43102600	-2.40730700
H	-1.54441100	-0.39955400	-2.47404200
C	-2.29242300	-3.60165100	-1.66176700
H	-1.80406300	-4.55942600	0.20390400
H	-2.69878500	-2.36874700	-3.37632300
H	-2.81209500	-4.46529900	-2.06050600
O	-3.11368700	0.55310200	0.39422300
C	-3.96544600	0.17331200	-0.50207800
C	-4.07790200	0.82494500	-1.76573700
C	-4.85544700	-0.91985400	-0.29318700
C	-4.98549300	0.40599800	-2.73046200
H	-3.43206200	1.67824900	-1.95234500
C	-5.75684100	-1.32771900	-1.26523000
H	-4.80279300	-1.43955000	0.65932300
C	-5.83400600	-0.67749200	-2.49969900
H	-5.03809300	0.93742500	-3.67757200
H	-6.41542700	-2.16800200	-1.06029800
H	-6.54581100	-0.99709400	-3.25257600
H	-3.43850800	4.00931800	2.65349700
O	-2.52655100	4.37260400	2.62880400
C	-2.15865600	4.85402900	3.83857000
C	-0.86400300	5.36297400	3.97243200
C	-3.01911000	4.86190900	4.94085700
C	-0.43880800	5.87023700	5.19336000
H	-0.20732500	5.35289000	3.11025100
C	-2.57941200	5.37227300	6.15804900
H	-4.02602600	4.47285700	4.83541500
C	-1.29041800	5.87875800	6.29619700
H	0.56830400	6.26378200	5.28330100
H	-3.25671100	5.37477300	7.00566200
H	-0.95521200	6.27679600	7.24682000
C	-5.38969800	2.02227700	2.17631000
C	-5.89157600	3.21122100	1.49202200
O	-5.05820500	3.34734200	2.65361200
H	-6.05799500	1.48147500	2.83990800
H	-4.56203400	1.47134700	1.73126500
H	-6.91781500	3.53253000	1.64284200
H	-5.42773900	3.50948700	0.55716300

TS_P2
Imaginary frequency value: -492.4
Sum of electronic and thermal Free Energies: -2035.232782

P	-2.14308600	0.90869700	0.87723400
C	-1.27874800	2.46090000	0.54633800
C	-1.71983600	3.66795600	1.09834600
C	-0.14428500	2.41623700	-0.27585200
C	-1.01665000	4.83719600	0.83400600
H	-2.60497500	3.70207000	1.72214100
C	0.54801300	3.59716200	-0.52319600
H	0.21347400	1.47450200	-0.70898700
C	0.11719900	4.80081000	0.02746600
H	-1.35497500	5.77491200	1.25933200
H	1.43247800	3.56830300	-1.14889500
H	0.66726300	5.71384800	-0.17086800
C	-3.72140900	1.26400900	1.71027600
C	-4.93749700	1.19605800	1.02743500
C	-3.69980400	1.62265900	3.06416700
C	-6.12221800	1.49663600	1.69283500
H	-4.96370500	0.89682400	-0.01344300
C	-4.88659300	1.92599000	3.71912800
H	-2.76172100	1.65282900	3.60751400
C	-6.09753100	1.86467300	3.03367800
H	-7.06482300	1.43653900	1.16133800
H	-4.86590100	2.20294600	4.76671900
H	-7.02272800	2.09663600	3.54881100
C	-1.20718200	-0.13765900	2.01331800
C	-1.84935100	-1.23084900	2.61149600
C	0.12885200	0.13940400	2.30634800
C	-1.14312400	-2.04228800	3.48888600
H	-2.89289100	-1.44118200	2.40336700
C	0.82976900	-0.67531300	3.19064000
H	0.62742600	0.98625700	1.85349900
C	0.19240700	-1.76414200	3.77634100
H	-1.63691000	-2.88948200	3.95096600
H	1.87306900	-0.45334800	3.39780300
H	0.73977800	-2.40084000	4.46233300
C	-2.46422000	0.09051200	-0.69712300
C	-2.97387100	0.85617800	-1.75514000
C	-2.22188400	-1.27278700	-0.86667400
C	-3.26680000	0.24280900	-2.96479900
H	-3.12024000	1.92506200	-1.64169900
C	-2.51076800	-1.87376300	-2.08654300
H	-1.77230200	-1.85472200	-0.07227900
C	-3.03947200	-1.12187300	-3.12864900
H	-3.65122800	0.83472400	-3.78730400
H	-2.28372600	-2.92184900	-2.23255000
H	-3.24443400	-1.59372000	-4.08214200
O	1.12771600	-0.23089300	-1.15821600
C	0.94849100	-1.00748800	-2.20108300
C	0.95126600	-2.42081400	-2.09866000
C	0.72477400	-0.46805100	-3.49108900
C	0.73656900	-3.22986100	-3.20786700
H	1.12887900	-2.86334700	-1.12354600
C	0.49976700	-1.28483000	-4.59114700
H	0.73455400	0.61201000	-3.60004100
C	0.50011500	-2.67475200	-4.46540800
H	0.75787200	-4.30998700	-3.09117600
H	0.33493900	-0.83267100	-5.56529500
H	0.34193400	-3.30936000	-5.33008400
H	4.23318200	-0.18674800	2.07030300
O	3.98457800	0.18425900	3.00050400
C	4.42957700	1.43918200	3.17271900
C	4.13042500	2.09998100	4.37206300
C	5.17985000	2.11269400	2.19689700
C	4.56449600	3.40239600	4.58477000
H	3.56366400	1.57017500	5.12982200
C	5.60701500	3.41610000	2.42401200
H	5.42327700	1.59949700	1.27334700
C	5.30412500	4.07449800	3.61368300
H	4.32679300	3.89522600	5.52227100
H	6.19070400	3.92059500	1.66041700
H	5.64511700	5.08912800	3.78476000
C	2.95297800	-0.24917100	-0.29177600
C	3.35585300	-1.52598300	0.28141800
O	4.43805000	-0.73118200	0.68395200
H	3.41984500	0.06609500	-1.21109400
H	2.53857500	0.50006600	0.37212500
H	3.61752900	-2.31911100	-0.42394900
H	2.72767800	-1.91450700	1.09163400

INT_P2
Sum of electronic and thermal Free Energies: -2035.289588

P	-1.91601400	0.98500600	1.88660800
C	-1.35597000	2.70859900	1.90110200
C	-2.29381400	3.74747300	1.98651700
C	0.00728200	2.99480800	1.75427100
C	-1.86238800	5.06732900	1.94815900
H	-3.35185700	3.53683400	2.07579000
C	0.42175700	4.32340100	1.72306500
H	0.76677300	2.21600400	1.64952500
C	-0.50588400	5.35498700	1.82262900
H	-2.58884700	5.86910200	2.01611400
H	1.48046000	4.53373900	1.62891400
H	-0.17161000	6.38626800	1.79938600
C	-3.70318200	0.98404200	2.22055100
C	-4.60527500	0.51954300	1.26216400
C	-4.17577700	1.46149700	3.45073800
C	-5.97100400	0.53163800	1.53223400
H	-4.24502000	0.15042500	0.30925400
C	-5.54539100	1.46997800	3.71199800
H	-3.48201600	1.82549500	4.20073100
C	-6.43753800	1.00480800	2.75318300
H	-6.66769600	0.17016400	0.78493700
H	-5.90132800	1.84014800	4.66419300
H	-7.50153000	1.01293600	2.96052500
C	-1.12609600	-0.08997900	3.11701200

C	-1.91940400	-0.88774400	3.95401400
C	0.27065000	-0.18662000	3.16273400
C	-1.31554000	-1.76172700	4.84959000
H	-2.99993100	-0.83835300	3.90864700
C	0.85556500	-1.06745000	4.06735700
H	0.94886500	0.38813600	2.52231300
C	0.07239300	-1.84931300	4.90962900
H	-1.93254500	-2.37517400	5.49620100
H	1.93690900	-1.13424000	4.09457400
H	0.54157100	-2.53318600	5.60832100
C	-1.63881400	0.28865000	0.24260800
C	-1.61420100	1.12658500	-0.87637400
C	-1.50613600	-1.09565200	0.09218000
C	-1.46047000	0.57447400	-2.14267400
H	-1.69360800	2.20112000	-0.75762500
C	-1.35656900	-1.63494200	-1.17880200
H	-1.49802300	-1.74259200	0.96200100
C	-1.33098900	-0.80304500	-2.29515700
H	-1.42078100	1.22314600	-3.00976300
H	-1.23384400	-2.70432900	-1.29993000
H	-1.18410700	-1.23068700	-3.28047700
O	2.83462800	-0.66832800	-3.72065300
C	1.94387100	-1.60061600	-4.12809800
C	1.28994000	-1.32819000	-5.33739400
C	1.65616900	-2.78986300	-3.44973800
C	0.36946600	-2.22640500	-5.85606100
H	1.52936400	-0.40620400	-5.85424200
C	0.73239200	-3.68471600	-3.98703400
H	2.13769500	-3.02367500	-2.50955200
C	0.08018200	-3.41535400	-5.18484200
H	-0.12127800	-2.00016500	-6.79682000
H	0.52838800	-4.60859600	-3.45544500
H	-0.63261100	-4.12089600	-5.59547400
H	2.96796500	0.01708900	0.57756800
O	2.47666900	1.15386400	1.60458100
C	3.28469100	2.11111200	1.98220400
C	3.24299200	2.64472700	3.29683600
C	4.22649900	2.69914100	1.09831300
C	4.06562500	3.69523400	3.68570700
H	2.54804700	2.20457300	4.00603100
C	5.04505200	3.74596600	1.49938300
H	4.29656900	2.30359000	0.09007400
C	4.97403900	4.26417300	2.79397400
H	4.00104600	4.07206700	4.70301400
H	5.75364900	4.16567500	0.79052900
H	5.61809700	5.08018800	3.10162700
C	3.47413800	-0.80513300	-2.44679200
C	2.57134800	-0.36102300	-1.30269000
O	3.19750400	-0.68752600	-0.09772000
H	3.81571500	-1.83265600	-2.28626300
H	4.35728300	-0.16790900	-2.50225700
H	1.59295700	-0.85818200	-1.40581400
H	2.39042400	0.71940800	-1.39203800

REA_B1
Sum of electronic and thermal Free Energies: -1026.826533

C	-1.49474900	-1.89790000	1.93542400
C	-2.63034800	-1.77978500	1.11282300
C	-1.30240900	-3.12514000	2.59730500
C	-3.53665800	-2.82440100	0.97169400
H	-2.82029400	-0.84696700	0.59256500
C	-2.18578000	-4.18557300	2.43123200
H	-0.43615100	-3.25233600	3.23820100
C	-3.31039200	-4.03361300	1.62401900
H	-4.41585800	-2.69715200	0.34969100
H	-2.00386400	-5.12772700	2.93708800
H	-4.00900300	-4.85489200	1.50487500
C	-0.18466900	0.24041300	0.90289900
C	0.09475900	1.60683700	1.09806300
C	-0.16803300	-0.23630300	-0.41984400
C	0.35678000	2.45668100	0.02650600
H	0.09481300	2.00936300	2.10591400
C	0.12961500	0.59977200	-1.49209300
H	-0.37753800	-1.28413000	-0.60737600
C	0.38355800	1.95071600	-1.27237800
H	0.55440300	3.50852500	0.20389100
H	0.15682200	0.20002100	-2.50015300
H	0.60318100	2.60736900	-2.10714900
C	0.23984400	-0.49531200	3.47700100
C	-0.39720000	-0.79283600	4.69608400
C	1.54953200	0.01553800	3.54247000
C	0.23229900	-0.57577400	5.91637300
H	-1.40929700	-1.18337900	4.68381700
C	2.19651900	0.20380600	4.75835600
H	2.07417400	0.25280700	2.62268600
C	1.53449800	-0.08382000	5.94926300
H	-0.28860200	-0.79518800	6.84213200
H	3.21316700	0.58132300	4.77988700
H	2.03193000	0.07520300	6.90003700
B	-0.48428300	-0.71852000	2.10935800
O	-3.08142000	2.86943800	0.87893300
C	-4.04250600	2.44513100	0.01633100
C	-3.75517800	1.67910900	-1.11430000
C	-5.36090100	2.80711300	0.29527200
C	-4.78801700	1.27906500	-1.95761500
H	-2.72851300	1.40166700	-1.33378200
C	-6.38131500	2.40120300	-0.55438700
H	-5.56446600	3.40194100	1.17760800
C	-6.10473400	1.63490000	-1.68531500
H	-4.55560200	0.68549400	-2.83531000
H	-7.40330900	2.68667100	-0.32995400
H	-6.90507200	1.32181200	-2.34529700
H	-2.21734100	2.53173600	0.61130900

TS_B1
Imaginary frequency value: -1281.4
Sum of electronic and thermal Free Energies: -1026.773034

C	-2.01343300	-1.32359500	1.26318800
C	-2.76257000	-1.61633800	0.11019500
C	-1.92490800	-2.33309200	2.23194900
C	-3.37874400	-2.84932500	-0.07250500
H	-2.88452900	-0.86296900	-0.66311300
C	-2.53625100	-3.57258700	2.05785600
H	-1.37281000	-2.14383100	3.14596000
C	-3.26407200	-3.83655300	0.90289500
H	-3.95442200	-3.03820400	-0.97248600
H	-2.44724200	-4.33062200	2.82904800
H	-3.74375300	-4.79961300	0.76489300
C	-0.12036800	0.43096600	0.20111200
C	0.77471400	1.50901100	0.37089200
C	0.20892500	-0.54375900	-0.76039500
C	1.94255100	1.60522400	-0.37114400
H	0.53884500	2.27318800	1.10431200
C	1.38201800	-0.45699600	-1.49813500
H	-0.45206100	-1.38929800	-0.91034800
C	2.24886100	0.61629100	-1.30417100
H	2.61693400	2.44087900	-0.22133700
H	1.62532700	-1.22655200	-2.22232000
H	3.16587600	0.68166700	-1.88012200
C	-0.66072100	0.42013600	2.90263200
C	-1.22210700	1.38070500	3.75263400
C	0.47736200	-0.25966300	3.36343100
C	-0.68488200	1.64149300	5.01210200
H	-2.08961700	1.94224000	3.42254900
C	1.02190100	-0.00366900	4.61707500
H	0.95366200	-0.99806200	2.72444000
C	0.43889500	0.94914800	5.44963200
H	-1.14347800	2.39046300	5.64952800
H	1.90382100	-0.54421500	4.94496300
H	0.86126300	1.15270400	6.42773800
B	-1.29758200	0.08721000	1.48042100
O	-2.24292900	1.33228900	1.00294700
C	-3.60328000	1.37959000	0.83783400
C	-4.11639300	2.09367600	-0.24368700
C	-4.46060800	0.78560800	1.76121900
C	-5.49299500	2.20879100	-0.40115100
H	-3.43363800	2.56058900	-0.94453400
C	-5.83561600	0.91011500	1.59245700
H	-4.05272400	0.22861200	2.59509600
C	-6.35935500	1.61784100	0.51449700
H	-5.88768300	2.76626700	-1.24344700
H	-6.50081300	0.44535700	2.31173200
H	-7.43198600	1.70843700	0.38931300
H	-1.33509400	1.01914900	0.19630900

INT_B1
Sum of electronic and thermal Free Energies: -1026.866979

C	-2.70165500	-1.89156500	2.41197900
C	-3.07541000	-2.36627100	1.14424800
C	-2.91499200	-2.73770100	3.51183200
C	-3.62616800	-3.63218200	0.98125300
H	-2.93097000	-1.74032500	0.27003100
C	-3.49274200	-3.99423000	3.35811200
H	-2.63510000	-2.40279800	4.50570100
C	-3.84354300	-4.44650500	2.08974200
H	-3.89524900	-3.98114700	-0.00979600
H	-3.66183700	-4.62289300	4.22572500
H	-4.28477500	-5.42977500	1.96462200
C	0.98662600	3.06238000	-2.84162600
C	0.52098200	3.55667000	-1.62583300
C	1.53390900	1.78379100	-2.90928400
C	0.60148100	2.77191400	-0.47827000
H	0.09654600	4.55339500	-1.57251800
C	1.61477300	0.99945800	-1.76138500
H	1.89790000	1.39918500	-3.85581100
C	1.14809500	1.49279400	-0.54572700
H	0.23881800	3.15479100	0.46910700
H	2.04149200	0.00372500	-1.81392600
H	1.20934300	0.88370900	0.34916200
C	-0.81710900	-0.23128800	3.54262300
C	-0.50514600	1.06694000	3.98092400
C	0.02619200	-1.27689100	3.95013100
C	0.59015900	1.30919700	4.80043900
H	-1.13772900	1.89192400	3.67118400
C	1.13248600	-1.03922200	4.75977700
H	-0.10915200	-2.28890500	3.61728400
C	1.41309800	0.25423900	5.19009100
H	0.80735100	2.31809600	5.13457800
H	1.77580200	-1.86103700	5.05526400
H	2.27201200	0.44116400	5.82580600
B	-2.04960800	-0.47907500	2.61038000
O	-2.50509200	0.64420800	1.96332200

C	-3.65389100	0.78378200	1.22375100
C	-3.54203700	1.26015800	-0.07929500
C	-4.90363400	0.53503300	1.78384600
C	-4.69330200	1.46792600	-0.83147700
H	-2.55845700	1.46233600	-0.48652600
C	-6.04758900	0.74747200	1.02173700
H	-4.97284600	0.17586600	2.80361500
C	-5.94876700	1.21181100	-0.28663400
H	-4.60566100	1.83517600	-1.84797900
H	-7.02101200	0.55064700	1.45702700
H	-6.84341800	1.37728400	-0.87558500
H	0.92462300	3.67384800	-3.73527300

REA_B2
Sum of electronic and thermal Free Energies: -1102.082710

C	-0.71805900	-1.78215000	0.42530100
C	-0.95447100	-2.48798500	-0.76567500
C	-1.00399800	-2.43363500	1.63604000
C	-1.44422400	-3.78843100	-0.74750200
H	-0.74715700	-2.01851400	-1.72125200
C	-1.52276400	-3.72430900	1.65892100
H	-0.82942800	-1.91791800	2.57516800
C	-1.73736100	-4.40676700	0.46535100
H	-1.60519900	-4.31851800	-1.67982700
H	-1.75116900	-4.20032200	2.60636800
H	-2.12992400	-5.41776800	0.47946800
C	0.96556000	0.14875900	1.43423000
C	1.14579500	1.51583800	1.70268000
C	1.82826500	-0.75994600	2.06929500
C	2.13496400	1.95759800	2.57403200
H	0.49693600	2.23637100	1.21650900
C	2.83460600	-0.32214700	2.92779700
H	1.71892100	-1.82257000	1.87841600
C	2.98552500	1.03841400	3.18483400
H	2.24994300	3.01752500	2.77364100
H	3.49744700	-1.04067200	3.39758500
H	3.76406700	1.38098500	3.85767500
B	-0.14192700	-0.32600700	0.42969800
O	-0.54720900	0.64612700	-0.44949500
C	-1.58859700	0.59908700	-1.34465500
C	-1.31155900	0.85295300	-2.68454900
C	-2.89561000	0.38585600	-0.91735000
C	-2.35315600	0.87231300	-3.60589200
H	-0.28666200	1.03197800	-2.98754800
C	-3.92905000	0.40851500	-1.84811700
H	-3.09513800	0.20164500	0.13139500
C	-3.66408000	0.64908000	-3.19314700
H	-2.13680200	1.06637400	-4.65051400
H	-4.94734200	0.23925900	-1.51627200
H	-4.47353300	0.66703900	-3.91352100
C	4.43115500	0.05787500	-1.35272300
C	5.22483900	-0.30260600	-2.44223100
C	3.83527100	1.31886000	-1.31226400
C	5.41717500	0.59764800	-3.48204800
H	5.67954200	-1.28592200	-2.45602800
C	4.03615500	2.21120700	-2.36113700
H	3.21682400	1.59832400	-0.46492500
C	4.82604000	1.85938600	-3.45083500
H	6.03609600	0.30979900	-4.32496800
H	3.56939600	3.18949100	-2.32077000
H	4.98108600	2.55817500	-4.26438400
O	4.27697800	-0.85843300	-0.35984000
H	3.69741800	-0.51120900	0.32937400

TS_B2
Imaginary frequency value: -1274.1
Sum of electronic and thermal Free Energies: -1102.019442

C	0.35137100	-1.84696900	-0.14966200
C	0.22025500	-2.48500300	-1.39395400
C	-0.19095200	-2.49560200	0.96379000
C	-0.40684600	-3.71969000	-1.51548100
H	0.60965500	-2.00249200	-2.28506000
C	-0.82568600	-3.73091100	0.85072300
H	-0.13316500	-2.02814200	1.94165000
C	-0.93161400	-4.34963900	-0.38971700
H	-0.49262200	-4.18886100	-2.49001300
H	-1.24014800	-4.20775000	1.73281900
H	-1.42478200	-5.31131800	-0.48128700
C	1.47328400	0.22412600	1.45695900
C	0.96142400	1.49807800	1.76889100
C	1.91085200	-0.59111700	2.52120900
C	0.86269700	1.92657000	3.08524900
H	0.62737800	2.14335700	0.96407000
C	1.81896800	-0.16391900	3.83893000
H	2.34061000	-1.56247300	2.29867100
C	1.28813400	1.09369200	4.11907600
H	0.45554400	2.90585800	3.31058300
H	2.16197000	-0.80171400	4.64587200
H	1.21168000	1.42945500	5.14780700
B	1.08779000	-0.43422000	-0.14187400
O	0.59980300	0.59214100	-0.98856700
C	-0.69403200	0.92889200	-1.22769800
C	-0.94946300	1.65839400	-2.39199300
C	-1.74403800	0.62452300	-0.35939700
C	-2.24206900	2.07046800	-2.68593300
H	-0.12168200	1.88557700	-3.05333300
C	-3.03669000	1.04028400	-0.66814700
H	-1.55633500	0.05872000	0.54377200
C	-3.29574100	1.76335100	-1.82691900
H	-2.42765300	2.63283100	-3.59469100
H	-3.84647900	0.79254300	0.00968100
H	-4.30466600	2.08340300	-2.05966600
C	3.23439300	-0.19909400	-1.71297700
C	3.62176400	-1.20322600	-2.59597900
C	3.47619500	1.13822700	-2.01486500
C	4.24590000	-0.86340000	-3.79186200
H	3.43605300	-2.23696500	-2.33075100
C	4.10519800	1.46685100	-3.21122400
H	3.16806300	1.91000800	-1.32040600
C	4.48964800	0.47085400	-4.10457400
H	4.54813000	-1.64696200	-4.47795300
H	4.29414500	2.50884600	-3.44499400
H	4.97941400	0.73276900	-5.03532900
O	2.65630200	-0.57540300	-0.52012000
H	2.41982600	0.10940400	0.52428300

INT_B2
Sum of electronic and thermal Free Energies: -1102.119527

C	0.30515800	-2.30008000	-0.95187600
C	-1.08924700	-2.44386300	-0.89543200
C	1.08823600	-3.24457000	-0.26839100
C	-1.67647400	-3.48454000	-0.18300600
H	-1.72938900	-1.74158800	-1.41618100
C	0.50597900	-4.27800600	0.45604800
H	2.16858000	-3.16424200	-0.31520200
C	-0.88062400	-4.39969300	0.49970000
H	-2.75650700	-3.58031600	-0.15949300
H	1.13111200	-4.99300000	0.98002700
H	-1.33881000	-5.20818900	1.05930600
C	2.16845000	0.24257200	2.77307200
C	2.38048600	1.51996500	3.28576400
C	0.92701700	-0.37131700	2.92171000
C	1.35110500	2.18442400	3.94740600
H	3.34791900	1.99677800	3.17133400
C	-0.10162800	0.29336000	3.58511200
H	0.76108400	-1.36486700	2.52047000
C	0.11018000	1.57060900	4.09792800
H	1.51644500	3.17903800	4.34692000
H	-1.06714500	-0.18624400	3.70341300
H	-0.69037700	2.08686900	4.61664600
B	1.02484900	-1.18011700	-1.77136300
O	0.47242300	-0.03004300	-2.26849300
C	-0.71593700	0.57028600	-1.94666700
C	-1.62663100	0.82174100	-2.96920800
C	-0.97478900	0.99451300	-0.64603000
C	-2.81520300	1.48330100	-2.67997700
H	-1.39205500	0.49807300	-3.97626700
C	-2.16768400	1.65432300	-0.36952900
H	-0.25164300	0.80696800	0.13893500
C	-3.09229700	1.90003200	-1.38065100
H	-3.52488500	1.67663300	-3.47671700
H	-2.36785900	1.98272400	0.64409400
H	-4.01821500	2.41805000	-1.15948900
C	3.17458000	-0.51649100	-2.72258500
C	3.76439200	-0.94033400	-3.90888300
C	3.47528200	0.73349400	-2.18994100
C	4.65326800	-0.10078300	-4.57175500
H	3.52153000	-1.92250200	-4.29666100
C	4.36487100	1.56559100	-2.86126600
H	3.01453900	1.04737000	-1.26126700
C	4.95598600	1.15465100	-4.05268500
H	5.11133300	-0.43207700	-5.49709800
H	4.59671600	2.54083300	-2.44774100
H	5.64943700	1.80691700	-4.57067400
O	2.35022700	-1.39530300	-2.05925100
H	2.97099700	-0.27609700	2.26000400

REA_B3
Sum of electronic and thermal Free Energies: -1177.337204

B	0.26315000	-1.13724900	1.32109900
O	1.11393700	-0.91842500	2.37277300
O	0.71413300	-1.95858500	0.32601700
C	2.41713900	-1.36494700	2.42154100
C	3.40407600	-0.72879400	1.67636200
C	2.73779000	-2.40829000	3.28215300
C	4.72345400	-1.15413200	1.78836000
H	3.13523600	0.09172000	1.02159900
C	4.06074700	-2.82547800	3.38712400
H	1.95184600	-2.87942800	3.86040800
C	5.05705400	-2.20249400	2.64117900
H	5.49714200	-0.66000700	1.20678500
H	4.31181200	-3.64030600	4.05707700
H	6.08686700	-2.52943100	2.72611900
C	0.20176500	-2.14971300	-0.93190400
C	0.10641900	-1.09119100	-1.83087300
C	-0.14656400	-3.44194500	-1.31375200
C	-0.36806800	-1.33215200	-3.11648200
H	0.39858500	-0.09373600	-1.52302900
C	-0.61689100	-3.66969400	-2.60281600
H	-0.04284600	-4.25061800	-0.60009800
C	-0.73213500	-2.61755000	-3.50751900
H	-0.44729700	-0.50691400	-3.81512700

H	-0.88978800	-4.67602400	-2.90055700
H	-1.09710400	-2.79914000	-4.51170000
C	-1.14267300	-0.45028900	1.41828600
C	-2.26638300	-0.87902500	0.69505500
C	-1.31031400	0.62108000	2.31370200
C	-3.50425700	-0.26533100	0.85956900
H	-2.18093700	-1.70519700	-0.00072900
C	-2.54259400	1.24899500	2.46756400
H	-0.46126100	0.95390400	2.90082400
C	-3.64417400	0.80306100	1.74125700
H	-4.36060500	-0.62037600	0.29691700
H	-2.64616400	2.07675700	3.16054800
H	-4.60890700	1.28291900	1.86628100
C	-0.57342600	3.05303900	-1.25391500
C	-1.96244000	3.16354100	-1.31627500
C	-2.54573900	3.97969800	-2.28077300
C	-1.75759200	4.68504900	-3.18431000
C	-0.37099600	4.56684500	-3.11410300
C	0.22451500	3.75729100	-2.15536100
H	-2.58134500	2.60985700	-0.61696400
H	-3.62644700	4.06070000	-2.32317600
H	-2.21662600	5.31949300	-3.93316400
H	0.25571000	5.11243700	-3.81109000
H	1.30178500	3.66205000	-2.08882300
O	0.05829100	2.26795700	-0.33747500
H	-0.58539400	1.86406500	0.25893900

TS_B3
Imaginary frequency value: -1211.8
Sum of electronic and thermal Free Energies: -1177.274004

B	-0.23471100	0.09051800	0.56290300
O	0.59262400	-0.51262900	1.53237000
O	-0.21848100	-0.31781000	-0.78671000
C	1.96073400	-0.46435200	1.41702100
C	2.67314300	0.44625400	2.19539500
C	2.63928400	-1.34677600	0.57805600
C	4.06221800	0.47286800	2.13273100
H	2.12863000	1.12514900	2.84040800
C	4.02952500	-1.31040400	0.52031500
H	2.07804400	-2.06003200	-0.01333000
C	4.74679000	-0.40315400	1.29470400
H	4.61064300	1.18367400	2.74127100
H	4.55274600	-2.00094300	-0.13225900
H	5.82952500	-0.38080900	1.24789600
C	-0.48185700	-1.56671400	-1.25184300
C	-0.49066400	-1.72703600	-2.64019200
C	-0.74482800	-2.66261300	-0.42617600
C	-0.75819200	-2.97092900	-3.19512400
H	-0.28420800	-0.86433200	-3.26262700
C	-1.01422600	-3.90407600	-0.99764800
H	-0.72782900	-2.54804300	0.65008200
C	-1.02305000	-4.06864500	-2.37812600
H	-0.76032900	-3.08293800	-4.27397800
H	-1.21634600	-4.74966500	-0.34900700
H	-1.23180300	-5.03889400	-2.81348300
C	-1.85839700	0.16374800	1.21536600
C	-2.96036600	0.04310800	0.34578500
C	-2.06659600	-0.05747200	2.59101300
C	-4.21665200	-0.29467100	0.82836900
H	-2.81588900	0.21035900	-0.71633500
C	-3.32036300	-0.39891100	3.07547300
H	-1.22353700	0.02618100	3.26752400
C	-4.39350800	-0.51769600	2.19255300
H	-5.05512400	-0.39227700	0.14831100
H	-3.46776200	-0.57539400	4.13502400
H	-5.37392300	-0.78720600	2.57082700
C	0.21604000	2.48015100	-0.51821500
C	-0.63164500	3.55736700	-0.76143000
C	-0.37097400	4.40957800	-1.82993500
C	0.73113200	4.19139800	-2.65059600
C	1.57575400	3.11460500	-2.39182300
C	1.32679500	2.25587100	-1.32735300
H	-1.48173400	3.72640600	-0.10998600
H	-1.03230100	5.24848600	-2.01654400
H	0.93346300	4.85690400	-3.48174600
H	2.44160000	2.94086600	-3.02127100
H	1.98264700	1.42107500	-1.11629800
O	-0.02165900	1.67633800	0.57364400
H	-1.18629500	1.28371400	0.96371500

INT_B3
Sum of electronic and thermal Free Energies: -1177.372676

B	1.36203500	-0.25907700	-0.40469900
O	2.11503100	-1.15478900	0.29843300
O	0.26210900	-0.68318300	-1.09028500
C	3.24699400	-0.85944400	1.02259700
C	3.28321100	-1.25325400	2.35601200
C	4.35203400	-0.25526100	0.43155300
C	4.43194100	-1.02727600	3.10655900
H	2.41363200	-1.73523200	2.78652800
C	5.49516900	-0.03264600	1.19217500
H	4.31281100	0.04200000	-0.60906400
C	5.54098400	-0.41535900	2.52951700
H	4.45867700	-1.33395400	4.14624300
H	6.35528300	0.44146300	0.73265800
H	6.43539400	-0.24106100	3.11618500
C	-0.17907200	-1.98780900	-1.15703100
C	-1.46496400	-2.26422300	-0.70680200
C	0.60762000	-2.98507400	-1.72351200
C	-1.96095900	-3.55928200	-0.81472000
H	-2.06236200	-1.46635800	-0.28237600
C	0.10115100	-4.27675300	-1.82452200
H	1.60572200	-2.75185200	-2.07386300
C	-1.18177200	-4.56954600	-1.37125700
H	-2.96326900	-3.77540800	-0.46235600
H	0.71496800	-5.05618000	-2.26209500
H	-1.57200600	-5.57732000	-1.45391400
C	-3.24074900	1.18138600	1.58820800
C	-3.42615900	1.41134300	0.22712100
C	-4.21844000	0.51582700	2.32326900
C	-4.59194500	0.97538800	-0.39782100
H	-2.66275700	1.92696000	-0.34445900
C	-5.38359600	0.08008700	1.69742500
H	-4.07299700	0.33734300	3.38314800
C	-5.57019900	0.30998300	0.33666900
H	-4.73671900	1.15381300	-1.45769800
H	-6.14573000	-0.43765100	2.26963700
H	-6.47812200	-0.02835700	-0.15074200
C	1.03548900	2.08164500	-1.04048700
C	0.73905100	3.22371400	-0.30264900
C	0.12273400	4.30359400	-0.92525800
C	-0.19367600	4.24816900	-2.27981300
C	0.11542400	3.10396700	-3.00945600
C	0.73331600	2.01787800	-2.39759100
H	1.00071800	3.25272600	0.74837100
H	-0.10784400	5.19198400	-0.34790500
H	-0.67310400	5.09108200	-2.76374300
H	-0.12255300	3.05254700	-4.06611800
H	0.97175000	1.12664400	-2.96387600
O	1.69671000	1.06582900	-0.39218300
H	-2.33337800	1.52127300	2.07530900
